# Identification of novel molecular regulators of tumor necrosis factor-related apoptosis-inducing ligand (TRAIL)-induced apoptosis in breast cancer cells by RNAi screening

**DOI:** 10.1186/bcr3645

**Published:** 2014-04-17

**Authors:** Sireesha V Garimella, Kristie Gehlhaus, Jennifer L Dine, Jason J Pitt, Magdalena Grandin, Sirisha Chakka, Marion M Nau, Natasha J Caplen, Stanley Lipkowitz

**Affiliations:** 1 Women's Maligancies Branch, Center for Cancer Research, 37 Convent Drive, Bethesda, MD 20892, USA; 2 Genetics Branch, Center for Cancer Research, National Cancer Institute, 37 Convent Drive, Bethesda, MD, 20892-4256, USA; 3 National Institute of Nursing Research, Bethesda, MD 20892, USA; 4 Laboratory of Cellular and Molecular Biology, Center for Cancer Research, National Cancer Institute, National Institutes of Health, 37 Convent Drive, Bethesda, MD 20892-4256, USA

## Abstract

**Introduction:**

Tumor necrosis factor-related apoptosis-inducing ligand (TRAIL) binds to its receptors, TRAIL-receptor 1 (TRAIL-R1) and TRAIL-receptor 2 (TRAIL-R2), leading to apoptosis by activation of caspase-8 and the downstream executioner caspases, caspase-3 and caspase-7 (caspase-3/7). Triple-negative breast cancer (TNBC) cell lines with a mesenchymal phenotype are sensitive to TRAIL, whereas other breast cancer cell lines are resistant. The underlying mechanisms that control TRAIL sensitivity in breast cancer cells are not well understood. Here, we performed small interfering RNA (siRNA) screens to identify molecular regulators of the TRAIL pathway in breast cancer cells.

**Methods:**

We conducted siRNA screens of the human kinome (691 genes), phosphatome (320 genes), and about 300 additional genes in the mesenchymal TNBC cell line MB231. Forty-eight hours after transfection of siRNA, parallel screens measuring caspase-8 activity, caspase-3/7 activity, or cell viability were conducted in the absence or presence of TRAIL for each siRNA, relative to a negative control siRNA (siNeg). A subset of genes was screened in cell lines representing epithelial TNBC (MB468), HER2-amplified breast cancer (SKBR3), and estrogen receptor-positive breast cancer (T47D). Selected putative negative regulators of the TRAIL pathway were studied by using small-molecule inhibitors.

**Results:**

The primary screens in MB231 identified 150 genes, including 83 kinases, 4 phosphatases, and 63 nonkinases, as potential negative regulators of TRAIL. The identified genes are involved in many critical cell processes, including apoptosis, growth factor-receptor signaling, cell-cycle regulation, transcriptional regulation, and DNA repair. Gene-network analysis identified four genes (*PDPK1*, *IKBKB*, *SRC,* and *BCL2L1*) that formed key nodes within the interaction network of negative regulators. A secondary screen of a subset of the genes identified in additional cell lines representing different breast cancer subtypes and sensitivities to TRAIL validated and extended these findings. Further, we confirmed that small-molecule inhibition of SRC or BCL2L1, in combination with TRAIL, sensitizes breast cancer cells to TRAIL-induced apoptosis, including cell lines resistant to TRAIL-induced cytotoxicity.

**Conclusions:**

These data identify novel molecular regulators of TRAIL-induced apoptosis in breast cancer cells and suggest strategies for the enhanced application of TRAIL as a therapy for breast cancer.

## Introduction

Tumor necrosis factor-related apoptosis-inducing ligand (TRAIL) may have potential use in cancer therapy because of its ability to kill selectively cancer cells over normal cells [1-3]. TRAIL binds to its receptors (TRAIL-R1 (DR4) or TRAIL-R2 (DR5)) on the cell surface, leading to the recruitment of the adaptor molecule FADD and pro-caspase-8 [4]. This forms the death-inducing signaling complex (DISC). Pro-caspase-8 is cleaved to its active form at the DISC, which then cleaves and activates the downstream executioners caspase-3 and caspase-7 (caspase-3/7), resulting in apoptosis. Active caspase-8 also can cleave the BH3 protein BID, resulting in activation of the intrinsic pathway of apoptosis by activating caspase-9 (reviewed in [5]). Studies in animals have shown that TRAIL mediates regression of cancer xenografts without affecting normal tissues, and human phase I studies have demonstrated that TRAIL agonists are safe [3,6]. However, the results published thus far have shown limited clinical efficacy, suggesting the need to identify predictive biomarkers that will stratify cancers into those more likely to respond and/or to identify additional genes or pathways that can be targeted in combination with TRAIL to enhance the efficacy of TRAIL agonists [6-14].

Preclinical studies have found that many cell lines of different cancer types are resistant to TRAIL. Initial studies of TRAIL-induced apoptosis in breast cancer cell lines demonstrated that although TRAIL could induce apoptosis in the MDA-MB-231 (MB231) breast cancer cell line, the majority of cell lines tested were very resistant to TRAIL-induced apoptosis [1,15-17]. These studies established that TRAIL induced caspase-mediated apoptosis in sensitive cell lines and that TRAIL activated caspases within minutes of addition to the cells [16,18]. Data from three independent studies, including ours, demonstrated that 10 of 14 triple-negative breast cancer (TNBC) cell lines were sensitive to TRAIL-induced apoptosis, whereas only two of eight HER2-amplified cell lines, and none of seven estrogen receptor (ER)-positive lines were sensitive to TRAIL-induced apoptosis [5,15,19,20]. Among the TNBC subtype, cells with mesenchymal features are more sensitive to TRAIL than are cells with epithelial features [20]. However, the underlying determinants of TRAIL sensitivity in the breast cancer cell lines have not been clearly established.

In this study, we took advantage of RNAi screening technology to identify novel molecular regulators of TRAIL-induced apoptosis in breast cancer cells. By using synthetic siRNA-mediated RNAi screens of the human kinome, phosphatome, and about 300 additional genes, we identified a subset of 150 genes that, when silenced, enhance TRAIL-induced caspase-3/7 activation in MB231 cells. These genes can be grouped into cellular networks that modulate the sensitivity to TRAIL in breast cancer cells. Analysis of the caspase-8 activation and cell viability RNAi screening data for those genes associated with these cellular networks corroborated a potential role for many of these proteins in regulating TRAIL-mediated apoptosis and cytotoxicity. RNAi screening of a subset of the identified genes in a panel of breast cancer cell lines representing different breast cancer subtypes (TNBC, HER2-amplified, and ER-positive) identified potential targets that may have broad application in enhancing TRAIL activity in breast cancer cells. Importantly, pharmacologic inhibition of two targets identified by RNAi screening, SRC or BCL2L1 (BCL-XL), sensitized cell lines known to be resistant to TRAIL-induced cell death, confirming the utility of the RNAi screen.

## Materials and methods

### Cell culture

The MB231, HCC38, BT549, BT474, MCF7, Hs578T, and SKBR3 breast cancer cell lines were obtained from ATCC; BT20 and HCC1937 were obtained from Reinhard Ebner (Avalon Pharmaceuticals; Germantown, MD, USA). All cells were grown in RPMI 1640 medium supplemented with 10% FBS and 1% Pen-Strep (R10). This research was performed with anonymized breast cancer cell lines and is exempt from ethics or IRB approval.

### Inhibitors and reagents

The GST-TRAIL construct and the isolation of recombinant GST-TRAIL fusion protein have been previously described [16]. The inhibitors PP2 (529573) and PP3 (529574) were obtained from Calbiochem (La Jolla, CA, USA), ABT-737 (S1002) was obtained from Selleck Chemicals (Houston, TX, USA), and DEVD-CHO (P410), from Biomol International (Plymouth Meeting, PA, USA). All inhibitors were dissolved in DMSO. Caspase-Glo 8 assay (G8202) and Caspase-Glo 3/7 assay (G8092), and Caspase-Glo 9 (G8210) systems were purchased from Promega Corporation (Madison, WI, USA).

### Caspase-activation assays, cell-viability assays, RNAi screening, and small-molecule-compound analysis

Primary RNAi screens were conducted by using siRNAs corresponding to 1,135 genes arrayed from the Human Druggable Genome Version 2.0 library (Qiagen Inc.). The siRNAs target 691 genes annotated as associated with kinase activity, 206 genes associated with phosphatase activity, and 238 additional genes that include members of the ABC transporter family and several apoptosis-associated genes. The majority of the genes within the kinase and phosphatase sets encode proteins with defined kinase or phosphatase activity, respectively, although a limited number act as enzyme co-factors, and a few have been re-annotated now as pseudogenes or withdrawn. See Additional file 1: Table S1 for full details of genes targeted. The 16 genes (four siRNAs per gene) selected for secondary screening are detailed in Additional file 2: Table S2.

For screening (primary and secondary), four siRNAs per gene were arrayed in 384-well plates, one siRNA per well. For each well, 2 pmol siRNA was complexed with 0.06 μl RNAiMax transfection reagent (Invitrogen; Grand Island, NY, USA) in 20 μl RPMI for 15 minutes at ambient temperature. Six hundred cells in 20 μl RPMI-1640/20% FBS were added to each well. Plates were maintained at room temperature for 15 minutes before incubation at 37°C/5% CO_2_. Paired screens were conducted: 48 hours after siRNA transfection, one screen received vehicle only (medium), whereas the other received 1,000 ng/ml TRAIL (in medium) for 1 hour for the study of caspase-3/7 and caspase-8 activation or 100 ng/ml of TRAIL (in medium) for 24 hours for the study of cell viability. The activation of caspase-8 and caspase-3/7 was measured by using Caspase Glo Assay systems following the manufacturer’s instructions (Promega Corporation with modification of the caspase-8 assay to block caspase-3/7-induced activation of caspase-8 (see Results for further details). Cell viability was measured by using Cell Titer Glo assay following the manufacturer’s instructions (G7572; Promega Corporation). All assay plates were measured with a Victor luminometer (Perkin Elmer, Waltham, MA, USA). The kinome and additional sets were screened together, whereas the phosphatase gene set was screened separately. As these screens were conducted at different times, the data for each screen was initially analyzed independently. To validate each screen, untransfected cells (cells only) and wells transfected with negative (AllStars Negative Control siRNA (siNeg); Qiagen, Valencia, CA, USA) and positive (AllStars Hs Celldeath siRNA (siCelldeath); Qiagen) control siRNAs were included on every plate, as were siRNAs corresponding to *CASP8* and *FLIP* (siCASP8, L003466 and siFLIP, L003772; from Dharmacon, Thermo Fisher Scientific, Waltham, MA). The data for each experimental siRNA were normalized by using the average value for siNeg-transfected cells without TRAIL for each plate. The data for all three screens are detailed in Additional file 1: Table S1.

For assay development and treatment with the SRC or BCL-XL inhibitors, cell viability was assessed by using the Cell Titer 96AQueous One Solution Cell Proliferation Assay (G3582) from Promega Corporation. All measurements were performed in replicates of six wells in a 96-well plate, and each experiment was carried out at least 3 times. Results are presented as the mean ± the standard error of the mean (SEM) of at least three independent experiments.

### Lysate preparation and immunoblotting

Cell lysates were made, and immunoblotting was performed as described earlier [20]. The following antibodies were used: anti-AKT (#4685), anti-phospho-AKT (T308; #4056), anti-caspase-8 (1C12; #9746), anti-ERK 1/2 (#9102), anti-phospho-ERK 1/2 (#9101), anti-GAPDH (#2118), anti-p70S6K (#2708), and anti-phospho-p70S6K (S371; #9205) from Cell Signaling Technology, anti-FLIP (#104) from Imgenex (San Diego, CA, USA), anti-SRC (#OP07) from EMD Millipore (Billerica, MA, USA), anti-phospho-SRC (#44-660G) from Life Technologies (Grand Island, NY, USA), and anti-Tubulin (#T9026) from Sigma Aldrich (St. Louis, MO, USA).

### Statistics and bioinformatics analysis

Student's *t* test (unequal variance) was used to determine statistical differences between siRNA control groups (calculated in Excel). A value of *P* < 0.05 was considered significant. A Pearson correlation coefficient was used to compare the relation between screens and was calculated in Excel. Paired Student's *t* tests were also performed to analyze the data for treatment with the SRC or BCL-XL inhibitors. To compare the effect of the combined treatment to the sum of the effects of the individual treatments, percentage inhibition was calculated for each condition as 100% viability. The inhibition of the combination was compared with the sum of the inhibition of TRAIL alone plus inhibitor alone. Knowledge-based gene networks were generated by using Ingenuity Pathway Analysis (IPA) tools (Ingenuity Systems; Redwood City, CA, USA).

## Results

### The development of assays for RNAi screens of TRAIL-induced apoptosis

**Figure 1:**
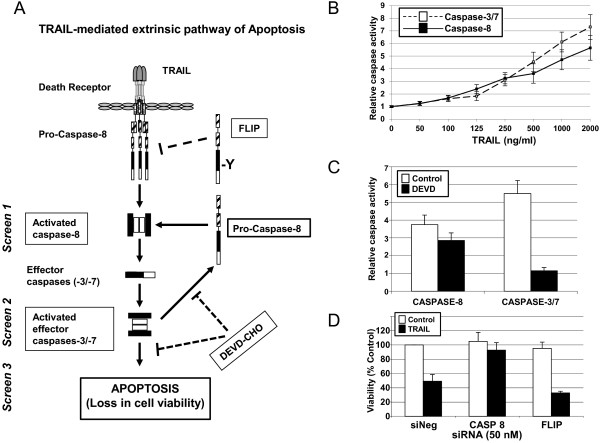
**The development of siRNA-based RNAi screens for the identification of regulators of TRAIL-induced apoptosis in the MB231 breast cancer cell line. (A)** A diagrammatic representation of the extrinsic TRAIL-induced apoptotic pathway. RNAi screens were developed assaying caspase-8 activation (Screen 1), caspase-3/7 activation (Screen 2), and cell viability (Screen 3) in the absence and presence of TRAIL. Synthetic siRNAs corresponding to *CASP8* and *FLIP* were used as positive and negative regulator controls of the TRAIL pathway, respectively. The caspase-3/7 DEVD-CHO inhibitor is shown on the diagram. **(B)** Caspase-3/7 and caspase-8 activity was measured by using caspase-3/7 and caspase-8-Glo assays. MB231 cells were treated with increasing concentrations of TRAIL (as indicated) or RPMI medium for 1 hour, after which caspase activity was measured. Fold-increase in caspase activity is plotted relative to the untreated cells. Data are shown as the mean and standard error of three experiments. **(C)** Caspase-3/7 and caspase-8 activity were measured after pretreatment with or without 0.03 μ*M* DEVD-CHO for 1 hour and then treatment with 1,000 ng/ml TRAIL for 1 hour. Inhibition with DEVD-CHO blocked caspase-3/7 activity significantly compared with caspase-8 activity. Data are shown as the mean and standard error of three experiments. **(D)** Viability of MB231 cells was measured by an MTS assay 48 hours after the transfection of the negative-control siRNA (siNeg) or siRNAs corresponding to *CASP8* and *FLIP,* respectively, either in the absence or presence of 1,000 ng/ml TRAIL for 17 hours. Data are shown as the mean and standard error of three experiments. **(E)** Western-blot analysis of cell lysates for CASP8 and FLIP expression, 48 hours after the transfection of the negative control siRNA (siNeg) or siRNAs corresponding to *CASP8* and *FLIP,* respectively.

To identify regulators of TRAIL-induced apoptosis, we established conditions compatible with siRNA-based RNAi screening for three assays that assess different steps in the TRAIL-induced apoptotic pathway in the MB231 breast cancer cell line. We chose to use the TRAIL-sensitive MB231 cell line and a concentration of TRAIL that induced approximately 50% maximum activity in each assay to enable identification of both positive and negative regulators of the TRAIL pathway. We used two assays that measured activation of caspases by TRAIL, one for activation of the initiator caspase-8, and one for the activation of the downstream effector caspases-3 and -7 (caspases-3/7). We also used an assay of cell viability (Figure 1A).

Assays were optimized to detect measurable levels of caspase-8 and caspase-3/7 activity by using substrates specific for each caspase. To identify an appropriate concentration of TRAIL to be used for identification of proteins that modulate early steps in TRAIL-induced apoptosis, MB231 breast cancer cells were treated with different concentrations of TRAIL and, after 1 hour, caspase activity was measured. A TRAIL concentration-dependent increase in activity was observed for both caspase-8 and caspase-3/7 (Figure 1B). At 1,000 ng/ml of TRAIL, we detected a sixfold change in caspase-3/7 activity and a 4.8-fold change in caspase-8 activity over untreated cells. The 1,000 ng/ml of TRAIL used to induce robust caspase activation within the 1-hour caspase assays is a much higher concentration than that needed to induce loss of viability when cells were exposed to TRAIL for >17 hours to assess cytotoxicity (discussed later).

Caspase-8 is the first caspase to be activated on TRAIL binding to its receptors. Also, caspase-8 can be activated in a retrograde fashion by active caspase-3/7 (Figure 1A) [21,22]. To measure the caspase-8 activity triggered by the TRAIL receptors and not that produced from active caspase-3/7, we treated cells with a caspase-3/7 inhibitor, DEVD-CHO, 1 hour before TRAIL treatment (Figure 1C). In the presence of 0.03 μ*M* DEVD-CHO, TRAIL-induced caspase-3/7 activity was inhibited to baseline levels in comparison with 5.5-fold activation over the untreated controls. By contrast, only a slight loss was found in TRAIL-induced caspase-8 activation in the presence of DEVD-CHO compared with TRAIL-induced activation of caspase-8 in the absence of DEVD-CHO (threefold versus 3.8-fold). Therefore, to ensure direct measurement of TRAIL-receptor-mediated caspase-8 activation, we used 0.03 μ*M* DEVD-CHO in our screening assay of caspase-8 activation.

To develop the screening assays further, we used control siRNAs corresponding to a positive effector of TRAIL-induced apoptosis, caspase-8 (CASP8), and a known negative regulator of TRAIL-induced apoptosis, the FLICE-like inhibitory protein (FLIP, *a.k.a.* CFLAR; [5]) (Figure 1A). Silencing of *CASP8* should lead to the suppression of apoptosis that can be assayed as an inhibition of caspase-8 and caspase-3/7 activation and a reduction in TRAIL-induced cytotoxicity. In contrast, silencing of *FLIP* should enhance the activation of caspase-8 and caspase-3/7 and further sensitize cells to TRAIL-induced cytotoxicity. We confirmed the effects of silencing *CASP8* and *FLIP* by transfecting MB231 cells with specific siRNAs for these genes and, 48 hours later, treating with 100 ng/ml of TRAIL. Control cells were transfected with a control siRNA (siNeg). Seventeen hours after the addition of TRAIL, cell viability was measured by MTS assay, and the values were plotted relative to untreated siNeg transfected cells (Figure 1D). In the siNeg-transfected control cells, treatment with TRAIL resulted in 49.0% ± 9.5% cell death. Caspase-8 is a known positive regulator of the TRAIL-induced apoptotic pathway, and its silencing resulted in decreased caspase activation similar to that of untreated cells. Upon silencing of *CASP8* and treatment with TRAIL, viability was 92.7% ± 10.45% and not statistically different from untreated *CASP8*-silenced cells (104.97% ± 12.73%). FLIP structurally resembles caspase-8, but lacks the proteolytic activity, and is a competitive inhibitor of the TRAIL pathway. The silencing of *FLIP* enhanced the sensitivity of MB231 cells to TRAIL, as measured by increased loss of cell viability (68.0% ± 2.0%) compared with siNeg-transfected cells. Thus, siRNAs corresponding to *CASP8* (siCASP8) and *FLIP* (siFLIP) were used as controls for positive and negative regulators of the TRAIL pathway, respectively, in all of the RNAi screens.

### RNAi screens of the TRAIL-induced apoptotic pathway in the breast cancer cell line MB231

**Figure 2:**
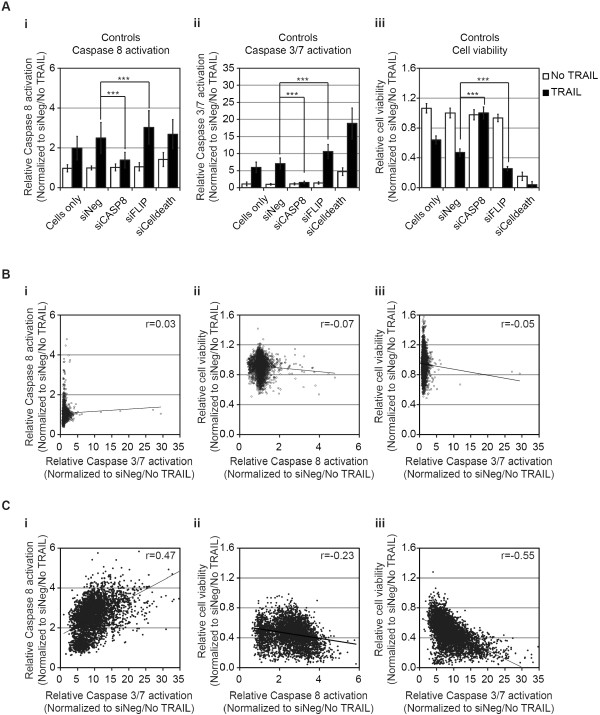
**Caspase-8, caspase-3/7, and cell-viability RNAi screens of TRAIL-induced apoptosis in MB231 cells. (A)** Controls included in the RNAi screens for (i) caspase-8 activation, (ii) caspase-3/7 activation, and (iii) cell viability in the absence (white bars) or presence (black bars) of TRAIL. For assessment of caspase-8 and caspase-3/7 activation, cells were siRNA-transfected and, 48 hours later, were treated with 1,000 ng/ml TRAIL for 1 hour. For assessment of cell viability, cells were siRNA-transfected, and 48 hours later were treated with 100 ng/ml TRAIL for 17 hours. Data are normalized to the mean value of negative control siRNA (siNeg)-transfected cells in the absence of TRAIL and are shown as the mean and standard deviation for each group by using the following number of wells: cells only, between 318 and 384 wells; and siRNA-transfected between 48 and 96 wells. Comparison of TRAIL-treated with siNeg-transfected untreated cells demonstrated a significant increase in caspase-8 and caspase-3/7 activation and a decrease in viability. siCASP8 reduced caspase-8 (*P* = 3 × 10^-21^) and caspase-3/7 (*P* = 2.5 × 10^-22^) activation and increased viability (*P* = 1.0 × 10^-48^) compared with siNeg-transfected cells. siFLIP significantly increased caspase-8 (*P* = 7.0 × 10^-5^) and caspase-3/7 (*P* = 2.3 × 10^-58^) activation and decreased viability (1.0 × 10^-67^) compared with siNeg-transfected cells. ***P < 0.001, by using a two-tailed Student's *t* test. **(B)** Scatterplots comparing results for each siRNA (total of 4,540) in each screen in the absence of TRAIL: (i) activation of caspase-3/7 versus caspase-8, (ii) activation of caspase-8 versus cell viability, and (iii) activation of caspase-3/7 versus cell viability. **(C)** Scatterplots comparing results for each siRNA (total of 4,540) in each screen in the presence of TRAIL: (i) activation of caspase-3/7 versus caspase-8, (ii) activation of caspase-8 versus cell viability, and (iii) activation of caspase-3/7 versus cell viability. The trend line for each data comparison is shown as the Pearson correlation (*r*).

RNAi screens designed to interrogate different aspects of the TRAIL-induced apoptotic pathway by measuring caspase-8 activation, caspase-3/7 activation, and cell viability were performed as described in the Materials and Methods. The kinome and additional gene sets were screened together by using all three-assay end points. The phosphatase gene set was screened separately by using just the caspase-3/7 activation and cell-viability assays. The kinome and additional gene sets were screened and analyzed together, whereas the phosphatase gene set was screened and analyzed separately. To validate each screen, wells of untransfected cells (cells only) and wells transfected with negative (siNeg) and positive (siCelldeath) control siRNAs were included on every plate, as were wells of siRNAs corresponding to *CASP8* and *FLIP*. A summary of the controls for the kinome/additional gene-set screen is shown in Figure 2A, and for the phosphatase gene-set screen, in Additional file 3, Figure S1A. The Z-factor values for each assay are shown in Additional file 4: Table S3.

In the absence of siRNA or in the siNeg-treated cells, TRAIL induced a twofold to 2.5-fold increase in caspase-8 activity and sixfold to sevenfold increase in caspase-3/7 activity (Figure 2Ai and 2Aii, respectively). Silencing of *CASP8* resulted in a significant reduction of TRAIL-induced caspase-8 and -3/7 activities, similar to the level of untreated cells (Figure 2Ai and 2Aii, respectively). Conversely, silencing of *FLIP* resulted in a statistically significant increase in caspase-8 or caspase-3/7 activity (Figure 2Ai and 2Aii, respectively). TRAIL induced an approximately 50% reduction in cell viability in untreated or siNeg-transfected cells (Figure 2Aiii). Silencing *CASP8* completely blocked the TRAIL-induced loss of viability, whereas silencing *FLIP* resulted in a significantly greater TRAIL-induced loss of viability (Figure 2Aiii). Similar results for caspase-3/7 activation and viability were seen in the control samples for the siRNA screen of the phosphatase gene set (Additional file 3: Figure S1A). The data for each experimental siRNA were normalized by using the average value for siNeg-transfected cells without TRAIL for each plate. The data for all three screens are detailed in Additional file 1: Table S1.

We first evaluated the correlation between the results for each siRNA in the three screens, a total of more than 4,000 data points (Figure 2B and C). In the absence of TRAIL, few siRNAs affected caspase-8 or caspase-3/7 activation or the viability of MB231 cells (Figure 2B). Although, for example, we did observe that three of four siRNAs corresponding to *PLK1* induced activation of caspase-8, caspase-3/7, and a decrease in viability in the absence of TRAIL (Additional file 3: Figure S1B), which is consistent with previous studies [23]. In contrast, in the presence of TRAIL, a substantial number of siRNAs increased activation of caspase-8 and caspase-3/7, and decreased the viability of MB231 cells in response to TRAIL (Figure 2C). Importantly, in the presence of TRAIL, a positive Pearson correlation of 0.47 was observed when levels of caspase-8 and caspase-3/7 were compared, whereas negative correlations were observed when caspase-8 activation or caspase-3/7 activation was compared with cell viability (caspase-8 versus cell viability: *r* = -0.23; and caspase-3/7 versus cell viability: *r* = -0.55). These data demonstrated that the effects on caspase activation and cell viability were generally consistent for each of the individual siRNAs.

### The identification of putative regulators of the TRAIL pathway

**Figure 3:**
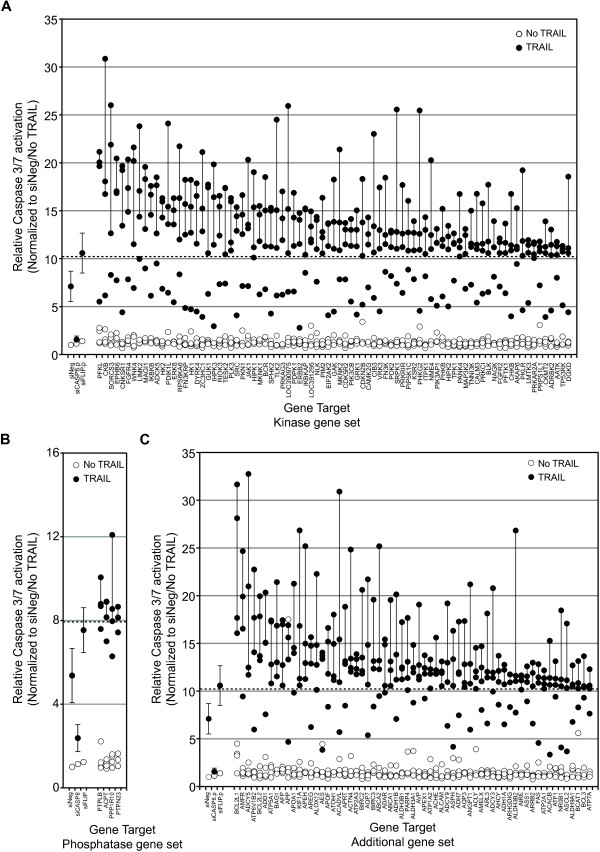
**The identification of putative negative regulators of TRAIL-induced apoptosis by using siRNA-enhanced activation of caspase-3/7 in the presence of TRAIL.** Genes for which three or four siRNAs mediated a fold change in the activation of caspase-3/7 in the presence of TRAIL of two or more standard deviations more than that observed in control siRNA (siNeg)-transfected cells plus TRAIL were considered putative negative regulators of TRAIL-induced apoptosis. The dashed line in each panel indicates the 2- standard deviation fold change, and a vertical line joins those siRNAs that induced at least this level of change for each gene. Data for the control siRNAs, siNeg, siCASP8, and siFLIP are included for reference and represent the same data for the siRNAs, indicated as shown in Figure 2Aii and Additional file 3: Figure S1Ai. **(A)** Genes identified from the kinase gene set. **(B)** Genes identified from the phosphatase gene set, and **(C)** genes identified from the additional gene set. Genes are ranked in descending order based on the median value for each set of four siRNAs per gene.

Of the three RNAi screening end points, overall, the siRNA screens of TRAIL conducted by using caspase-3/7 as an end point showed the greatest range of fold-change in activation relative to controls (up to >30-fold). Thus we chose to focus on the results of the caspase-3/7 screen to initially identify regulators of TRAIL and use the caspase-8 and cell-viability screening data to corroborate our findings. We defined putative negative regulators of TRAIL as those genes for which at least three of the four siRNAs tested caused an increase in TRAIL-induced caspase-3/7 activation two standard deviations or more from the TRAIL-induced caspase-3/7 activation, seen with the control siNeg siRNA. This corresponded to a >10.28-fold change for the kinase and additional gene set screens and a >7.96-fold change for the phosphatase gene set. These fold changes were comparable with that seen after silencing of the negative regulator *FLIP* (10.58-fold change for the kinase and additional gene-set screens and 7.54-fold for the phosphatase gene set). These criteria identified 83 kinases or kinase-related genes (Figure 3A), four phosphatases (Figure 3B), and 63 genes from the additional gene set (Figure 3C), whose silencing augmented TRAIL-induced caspase-3/7 activity. The screen identified several known negative regulators of apoptosis as negative regulators of TRAIL-induced caspase-3/7 activation, including *BCL2L1* (BCL-XL), *BCL2L2* (BCL-w), *BIRC2* (c-IAP1), and *BIRC3* (c-IAP2) [24,25].

Also we assessed whether any genes act as positive regulators of TRAIL activity. We defined positive regulators of TRAIL-induced caspase activation as those genes in which at least three of four siRNAs resulted in TRAIL-induced caspase-3/7 activation that was two or more standard deviations less than that seen in cells treated with the siNeg control (a <4.06-fold change for the kinase and additional gene-set screens and a <2.77-fold change for the phosphatase gene set). Interestingly, with these criteria, no positive regulators of TRAIL-induced caspase-3/7 activation were identified. Silencing of *CASP8* clearly inhibited TRAIL-induced activation of caspase-3/7 by more than 2 standard deviations, indicating that the screen was capable of identifying such genes (Figure 2Aii). Relaxing the criteria to siRNAs that resulted in more than a 1 standard deviation reduction in TRAIL-induced caspase-3/7activation compared with the siNeg-control, identified eight genes as putative positive regulators of TRAIL-induced caspase-3/7 activation (*NEK6*, *ETNK1*, *NME5*, *PXK*, *CALM2*, *RPS6KB2*, *GK5*/*MGC40579*, and *AKR1B1;* Additional file 3: Figure S1C).

### Gene-network analysis and experimental corroboration of negative regulators of TRAIL

**Figure 4:**
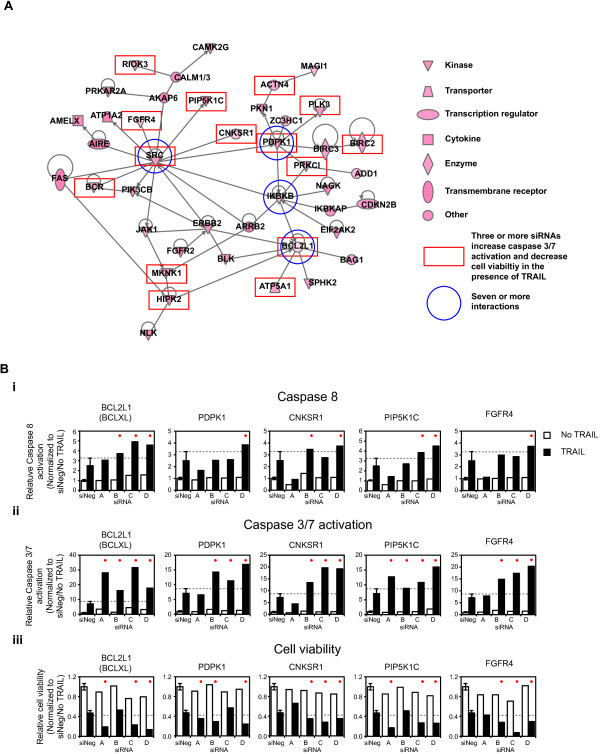
**Network analysis of putative negative regulators of TRAIL-induced apoptosis. (A)** A gene-interaction network generated by Ingenuity Pathway Analysis of the 150 genes for which three or more siRNAs induced increased TRAIL-induced activation of caspase-3/7 levels. The blue circles indicate those genes with seven or more interactions, and the red rectangles, genes for which three or more siRNAs caused both an increase in TRAIL-induced activation of caspase-3/7 levels and decreased viability in response to TRAIL. Legend shows the symbols indicated by the Ingenuity Pathway Analysis software. **(B)** Assays of (i) caspase-8, (ii) caspase-3/7 activation, and (iii) cell viability in the absence (empty bars) and presence (black bars) of TRAIL for four siRNAs (A-D) corresponding to the genes shown. Average data (±1 standard deviation) for negative control siRNA (siNeg)-transfected cells is shown in each graph. The grey dashed line indicates the relevant 1 standard deviation value for each assay, and the red dots indicate those siRNAs inducing fold-changes greater than 1 standard deviation.

We focused our subsequent analysis on putative negative regulators of TRAIL-induced caspase-3/7 activation rather than on positive regulators because of the number of genes identified and because they may be potential targets that, when inhibited, will enhance TRAIL-induced apoptosis. Given the relatively large number of putative negative regulators of TRAIL-induced apoptosis, we subjected the 150 genes to network gene analysis to aid in identification of common regulatory networks in which these genes function. Of the 147 genes with curated interaction data, the largest network identified connected 79 genes (see Additional file 5: Figure S2). Of these 79 genes, 42 were connected principally *via* four genes with seven or more interactions (Figure 4A). The genes situated at these nodes are *BCL2L1* (BCL-XL), *IKBKB, PDPK1,* and *SRC* (indicated by the blue circles in Figure 4A).

To investigate further the TRAIL-associated loss-of function (LOF) phenotype of the 79 genes that formed this regulatory network, we examined the results of silencing these genes on caspase-8 activation and cell viability in the presence of TRAIL. Three or more siRNAs against 26 of the 79 genes that enhanced TRAIL-induced activation of caspase-3/7 also enhanced TRAIL cytotoxicity by greater than 2 standard deviations from the mean viability seen in siNeg-transfected cells plus TRAIL. As indicated by the red rectangles in Figure 4A, 14 of these 26 genes map to the direct interaction network. The silencing of *BCL2L1* and two genes directly linked to it, *ATP5A1* and *HIPK2*, by multiple siRNAs increased TRAIL-induced caspase-3/7 activation and cytotoxicity (Figure 4B and Additional file 6: Figure S3). In addition, the RNAi induced LOF of several genes linked to *SRC* enhanced TRAIL-induced cytotoxicity including *PDPK1*, *CNKSR1*, *PIP5K1C*, *FGFR4*, *BCR, RIOK3,* and *MKNK1* (Figure 4B and Additional file 6: Figure S3). All three of the siRNAs corresponding to *SRC* that activated caspase-3/7 in the presence of TRAIL enhanced cytotoxicity, but only by using a relaxed criterion of greater than 1 standard deviation from the mean viability seen in siNeg-transfected cells plus TRAIL (Additional file 6: Figure S3). Multiple siRNAs corresponding to *PDPK1* and several genes linked to *PDPK1* (Figure 4B) also increased TRAIL-induced caspase-3/7 activation and cytotoxicity. These included *PRKC1*, the known apoptosis inhibitor *BIRC2* (*a.k.a.*, *cIAP-1*), *PLK3*, *PKN1*, and *ACTN4* (Additional file 6: Figure S3). Silencing by two of four siRNAs of many of the remaining genes mapping to the direct interaction network induced a decrease in cell viability greater than 2 standard deviations from that seen in siNeg-transfected cells plus TRAIL, with at least one further siRNA inducing a decrease in viability at least 1 standard deviation from that seen in siNeg-transfected cells plus TRAIL. This included silencing of *IKBKB* (Additional file 6: Figure S3), *BLK*, *ERBB2*, *FGFR2*, *NAGK,* and *ZC3HC1 (PARP12)*.

The results for the activation of caspase-8 were more variable. Only one gene *BCL2L1* (BCL-XL) showed an increase in caspase-8 levels more than 1 standard deviation from that seen in siNeg-transfected cells for three or more siRNAs (Figure 4Bi). In several other cases, two of four siRNAs corresponding to a specific gene mediated an increase in caspase-8 levels more than 1 standard deviation from that seen in siNeg, including *CNKSR1*, *BCR,* and *PIP5KIC,* which all linked to *SRC* (Figure 4Bi and Additional file 6: Figure S3), *ATP5A1* that links to *BCL2L1* (Additional file 6: Figure S3)*,* and *PRKC1,* which is linked to *PDPK1* (Additional file 6: Figure S3). Two genes for which three siRNAs activated caspase-3/7 and -8 but did not map to the network based on direct interactions, *BCL2L2* and *APEX1*. Interestingly, all four siRNAs corresponding to *BCL2L2* (BCL-w) enhanced TRAIL-induced caspase-3/7 activation, and three of these siRNAs also enhanced TRAIL-induced caspase-8 activation, but no effect on cell viability was observed (Additional file 6: Figure S3). Three siRNAs corresponding to the APEX nuclease (multifunctional DNA repair enzyme) 1 gene, *APEX1*, enhanced TRAIL-activated caspase-3/7 and caspase-8 and decreased cell viability, but the individual siRNAs that generated these phenotypic changes were inconsistent (Additional file 6: Figure S3).

### Secondary RNAi screen validation in additional breast cancer cell lines

To validate a subset of the genes identified by the primary siRNA screen in MB231 as putative negative regulators of TRAIL activity, we selected 16 genes for secondary screening in four breast cancer cell lines and assayed caspase-3/7 activation. Fifteen of the genes chosen (annotated by red boxes in Figure 4) were those that, when silenced, induced both an increase in activation of caspase-3/7 and a decrease in viability in response to TRAIL (Figures 4 and Additional file 6: Figure S3). We also included *IKBKB,* as this formed a node in the interaction map with seven or more interactions, and when silenced, three of four siRNAs induced an increase in TRAIL-induced activation of caspase-3/7, and two of four siRNAs decreased viability in response to TRAIL (Figures 4 and Additional file 6: Figure S3).

For the secondary screen, we used MB231 and three additional breast cancer cell lines (MB468, SKBR3, and T47D) representing different subsets of breast cancer with different sensitivities to TRAIL. The MB231 cell line is a basal B/TNBC cell line, MB468 is a basal A/TNBC cell line, SKBR3 is a HER2 amplified cell line, and T47D is an ER-positive cell line [20,26]. Upon treatment with TRAIL, a robust activation of caspase-3/7 occurs in the MB231 cell line, an intermediate activation of caspase-3/7 in the MB468 cell line, and little or no caspase-3/7 activation in the SKBR3 and T47D cell lines (see Additional file 7: Figure S4). The siRNAs used for the secondary screen are listed in Additional file 2: Table S2. Some of the siRNAs used in the primary screen were no longer available, and substitutes were obtained.

**Figure 5:**
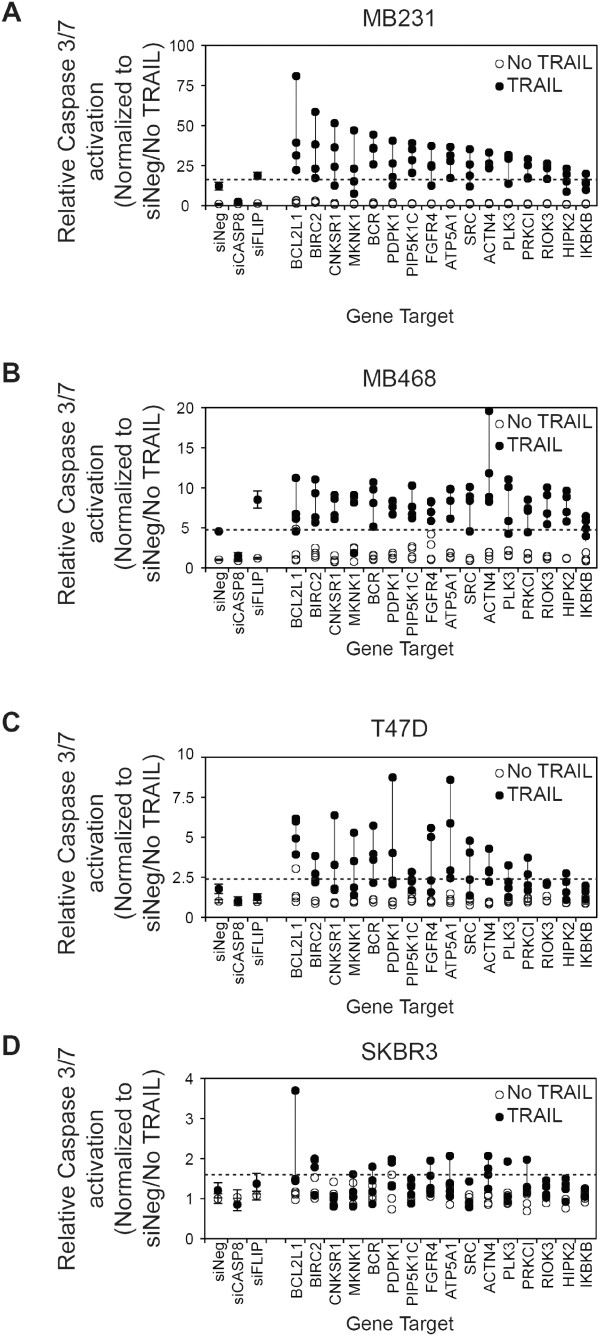
**A secondary screen of putative negative regulators of TRAIL-induced apoptosis.** Four siRNAs corresponding to 16 genes were selected for additional analysis of TRAIL-induced caspase-3/7 activation in **(A)** MB231, **(B)** MB468, **(C)** T47D, and **(D)** SKBR3. Cells were siRNA transfected, as for the primary screen, and 48 hours later were treated with 1,000 ng/ml TRAIL for 1 hour before assessment of caspase-3/7 activation. The dashed line in each panel indicates the 2-standard deviation fold-increase in TRAIL-induced caspase-3/7 activation compared with TRAIL-treated cells transfected with negative control siRNA (siNeg). The vertical line joins the siRNAs for each gene. Genes are ranked in descending order based on the median value for each set of four siRNAs per gene in the MB231 cell line. Data for the control siRNAs, siNeg, siCASP8, and siFLIP are included for reference.

The results for the secondary screen are detailed in Figure 5 and Additional file 8: Table S4 and summarized in Table 1. Upon rescreening the 16 genes in MB231, the silencing of 13 of the 16 genes again showed a 2-standard deviation increase in TRAIL-induced caspase-3/7 activity by three or more of the siRNAs to each target (Figure 4A and Table 1 and Additional file 8: Table S4). We used two criteria to rank the degree of validation of a gene as a negative regulator of TRAIL-induced apoptosis based on three or more siRNAs corresponding to each gene enhancing TRAIL-induced caspase-3/7 activation by either (a) greater than 2 standard deviations (indicated in Table 1 as a +; high stringency) or (b) greater than 1 standard deviation (indicated in Table 1 as a (+); low stringency) from that observed in siNeg-transfected cells treated with TRAIL.

**Table 1:** Summary of secondary screen of TRAIL-induced caspase-3/7 activation

**Gene**	**MB231 (TNBC/Basal B)**	**MB468 (TNBC/Basal A)**	**T47D (ER+)**	**SKBR3 (HER2+)**
**ACTN4**	**+**	**+**	**+**	**+**
**BCL2L1**	**+**	**+**	**+**	**(+)**
**BIRC2**	**+**	**+**	**(+)**	**+**
**ATP5A**	**+**	**+**	**+**	**-**
**BCR**	**+**	**+**	**+**	**-**
**FGFR4**	**+**	**+**	**(+)**	**-**
**PDPK1**	**+**	**+**	**(+)**	**-**
**PIP5K1C**	**+**	**+**	**(+)**	**-**
**RIOK3**	**+**	**+**	**(+)**	**-**
**SRC**	**+**	**+**	**(+)**	**-**
**CNKSR1**	**+**	**+**	**-**	**-**
**HIPK2**	**(+)**	**+**	**-**	**-**
**MKNK1**	**(+)**	**+**	**-**	**-**
**PLK3**	**+**	**+**	**-**	**-**
**PRKC1**	**+**	**+**	**-**	**-**
**IKBKB**	**-**	**+**	**-**	**-**

**+** indicates that three or more siRNAs for a target enhanced TRAIL-induced caspase-3/7 activity by >2 standard deviations (criterion 1).

(**+**) indicates three or more siRNAs for a target enhanced TRAIL-induced caspase-3/7 activity by >1 standard deviation (criterion 2).

In MB231 cells, 13 of the 16 genes were validated at high stringency (based on criterion 1). LOF of two genes, *MNNK1* and *HIPK2*, only replicated when a more-relaxed stringency (criterion 2) was used (Figure 5A, Table 1, and Additional file 8: Table S4). Only LOF of IKBKB failed to replicate based on the lower stringency although two of the four siRNAs increased TRAIL-induced caspase-3/7 activation by more than 1 standard deviation. Overall, these results in MB231 confirmed the reliability of the primary screen results. Interestingly, LOF of all 16 genes enhanced TRAIL-induced caspase-3/7 activation in the TNBC/basal A cell line MB468 by using the high-stringency criterion of three or more siRNAs enhancing TRAIL-induced caspase-3/7 activation by more than 2 standard deviations. (Figure 5B, Table 1, and Additional file 8: Table S4).

The ER-positive T47D cell line and the HER2-amplified cell line SKBR3 are resistant to TRAIL-induced cytotoxicity (Additional file 7: Figure S4). Any alteration in the sensitivity of these cells to TRAIL is likely to represent an important regulator of TRAIL and a potential target for enhancing its activity in breast cancer more broadly. In T47D cells, the LOF of four genes met the high-stringency criterion 1 (*BCL2L1, BCR, ATP5A1,* and *ACTN4*), and six additional genes met the lower-stringency criterion 2 (*BIRC2, PDPK1, PIP5K1C, FGFR4, SRC,* and *RIOK3*) (Figure 5C, Table 1, and Additional file 8: Table S4). In SKBR3, the LOF of only two genes met the high-stringency criterion (*BIRC2* and *ACTN4*), and the LOF of one additional gene (*BCL2L1*) met the lower-stringency criterion 2 (Figure 5D, Table 1, and Additional file 8: Table S4). Overall, this suggests that *BCL2L1, BIRC2,* and *ACTN4* are potentially major regulators of TRAIL-induced caspase-3/7 in breast cancer and that their LOF has the potential to overcome resistance to TRAIL-induced cytotoxicity. Other genes, including *ATP5A*, *BCR*, *FGFR4*, *PDPK1*, *PIP5K1C, RIOK3*, and *SRC*, also act to regulate TRAIL-induced apoptosis, but their potential to overcome resistance to TRAIL-induced cytotoxicity when inhibited may be more context specific (that is, in a more-restricted subset of breast cancer cells).

### The inhibition of SRC or BCL-XL enhances TRAIL sensitivity of TRAIL-resistant breast cancer cell lines

To translate the results of the RNAi screens by using a pharmacologic approach, we chose next to focus on *SRC* and *BCL2L1* (BCL-XL), for which small-molecule inhibitors are readily available. Based on our siRNA studies, the LOF of SRC may potentially represent a context-specific modulator of TRAIL activity, whereas LOF of BCL2L1 may modulate TRAIL activity in a broader range of breast cancer cell types.

**Figure 6:**
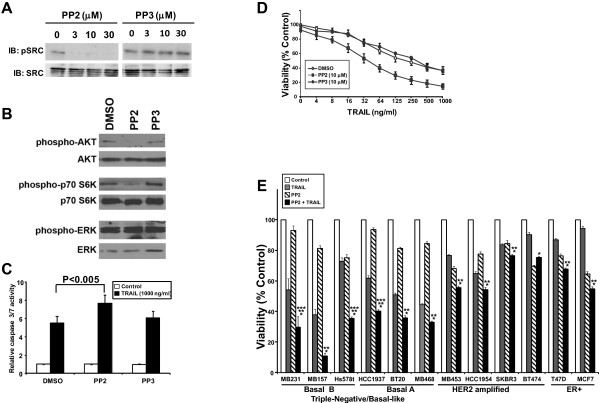
**Inhibition of SRC sensitizes breast cancer cells to TRAIL. (A)** Western blot analysis of MB231 cell lysates treated with different concentrations of SRC inhibitor PP2 or its inactive analogue PP3 for 1 hour, probed for phospho-SRC (pSRC) and total SRC. **(B)** Western blot analysis of MB231 cell lysates treated with 10 μ*M* PP2, PP3, or DMSO for 4 hours and probed for the indicated proteins. **(C)** Caspase-3/7 activation by pretreatment with PP2 or PP3 for 4 hours followed by the addition of DMSO (white bars) or 1,000 ng/ml of TRAIL (black bars) for 1 hour. Data are the mean ± SEM for three experiments. The comparison between PP2-treated and DMSO-treated cells was performed by using a paired, two-tailed Student's *t* test. **(D)** Cell-viability assay of MB231 cells treated with 10 μ*M* PP2 or PP3 or DMSO for 1 hour followed by different concentrations of TRAIL treatment for 17 hours. **(E)** Cell viability of different breast cancer cells after pretreatment with or without 10 μ*M* PP2 for 1 hour followed by treatment with 125 ng/ml TRAIL for 17 hours, as measured by MTS assay. Data are shown as the mean ± SEM for three experiments for each cell line, and the data were compared by using a two-tailed Student's *t* test. *P < 0.05, comparing the combination treatment with TRAIL alone; **P < 0.05 comparing the combination treatment with PP2 alone; and ***P < 0.05 comparing the combination treatment with the sum of the inhibition by TRAIL alone plus the inhibitor alone.

In our screen of additional breast cancer cell lines, the LOF of SRC enhanced TRAIL-induced caspase-3/7 activation by 2 or more standard deviations in the two TNBC cell lines MB231 and MB468, and by 1 standard deviation in the ER-positive cell line T47D (Figure 5, Table 1, and Additional file 8: Table S4). Inhibition of SRC in MB231 cells by the SRC-kinase family small-molecule inhibitor, PP2, resulted in decreased autophosphorylation of SRC compared with its nonfunctional structural analogue, PP3 (Figure 6A) [27]. Prior work demonstrated that inhibition of SRC led to decreased activation of the PI3 kinase/AKT pathway, and this, in turn, resulted in increased TRAIL sensitivity [28,29]. To test this, we examined the effects of PP2 on downstream signaling pathways and demonstrated that treatment with PP2 results in a decrease in activated AKT and activated p70 S6 kinase, as measured by phosphorylation of these proteins (Figure 6B). By contrast, no effect was seen in phosphorylation of ERK (Figure 6B).

Silencing of SRC by RNAi followed by TRAIL treatment enhanced caspase-3/7 activation by more than 10-fold over siNeg-treated cells (Figure 3A and Additional file 1: Table S1). To test whether PP2 has similar effects, we treated MB231 cells with PP2 or PP3 for 2 hours followed by 1,000 ng/ml TRAIL, and measured caspase-3/7 activation (Figure 6C). Cells treated with TRAIL exhibited a sixfold increase in caspase-3/7 activity over untreated cells. Inhibition of SRC by PP2 followed by TRAIL treatment resulted in a 40% increase in the caspase-3/7 activity over control cells. Cells treated with PP3 and TRAIL showed no significant increase in caspase-3/7 activation compared with control cells.

The effect of inhibiting SRC on TRAIL-induced loss of viability was tested in MB231 cells preincubated with either PP2 or PP3 before the addition of TRAIL (Figure 6D). The IC_50_ of TRAIL in these cells was about 125 ng/ml; the SRC inhibitor, PP2, by itself did not affect cell viability at 10 μ*M* (92.9% ± 2.3%), but the sensitivity of the cell line to TRAIL was significantly enhanced in the presence of PP2 with an IC_50_ for TRAIL of approximately 32 ng/ml in the presence of PP2 (*P* < 0.05). The inactive compound, PP3, had little or no effect alone or in combination with TRAIL.

Previously we showed that TNBC cells are more sensitive to TRAIL than are other subtypes of breast cancer [20]. We next investigated whether SRC inhibition would sensitize TRAIL-resistant cells to TRAIL by testing the combination of TRAIL ± PP2 on a panel of breast cancer cell lines representing ER-positive (T47D or MCF7), HER2 amplified (SKBR3, BT474, HCC1954, MB453), TNBC/basal A (HCC1937, BT20, MB468), and TNBC/basal B (MB231, MB157, Hs578t) subtypes (Figure 6E). The combination of TRAIL and PP2 was more effective than TRAIL alone in all cell lines tested (*P ≤ 0.05) and was more effective than PP2 alone in all cell lines except the HER2-amplified cell line BT474 (**P ≤ 0.05). When the inhibition of viability by the combined treatment was compared with the sum of the inhibition seen with TRAIL alone and PP2 alone, a significant difference was seen in the TNBC/basal B cell lines MB231 and Hs578t, and the TNBC/basal B cell line HCC1937 (***P ≤ 0.05). Although the combination appeared more active than the sum of the two agents alone in the TNBC/basal B cell line MB157, these data did not reach statistical significance, in part because of the high sensitivity to TRAIL alone in this cell line. In the other cell lines, although the combination was more toxic than either treatment alone, the effects were relatively modest and not greater than the sum of the individual treatments (Figure 6E).

Our primary screen identified *BCL2L1* (BCL-XL) and *BCL2L2* (BCL-w), known negative regulators of the mitochondrial (intrinsic) apoptosis pathway, as putative negative regulators of TRAIL-induced apoptosis in MB231 cells (Figure 3). Further, *BCL2L1* was identified as a node in the gene-interaction network generated by using our RNAi screening data (Figure 4). Silencing of *BCL2L1* enhanced TRAIL-induced caspase activation in three of the four cell lines tested at high stringency (2-standard deviation cutoff) and in all four lines if a lower stringency was used (Figure 5, Table 1, and Additional file 8: Table S4).

**Figure 7:**
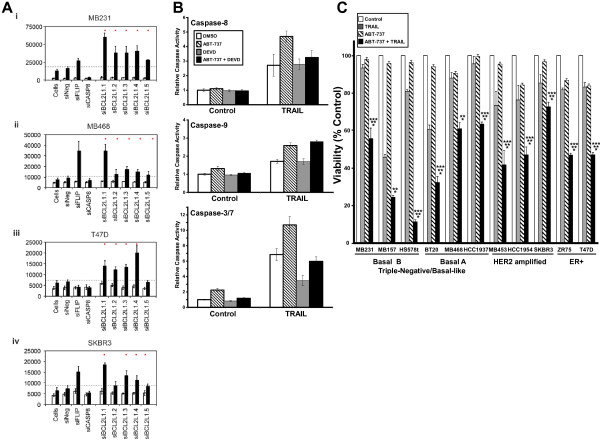
**Inhibition of BCL2L1 (BCL-XL) sensitizes breast cancer cells to TRAIL. (A)** MB231, MB468, T47D, and SKBR3 cells were incubated with five different targeting siRNAs for 48 hours, and caspase-3/7 activity was assayed 1 hour after addition of 1,000 ng/ml TRAIL. siNeg, siFLIP, and siCasp8 are included in each screen as controls. The grey dashed line represents 1-standard deviation increase in TRAIL-induced caspase-3/7 activation compared with TRAIL-treated cells transfected with the siNeg control. The red dots indicate those siRNAs that induced a fold-change greater than 1 standard deviation. **(B)** MB231 cells were incubated with TRAIL (1,000 ng/ml), the BCL2 family inhibitor ABT-737 (5 μ*M*), or both in the presence or absence of DEVD-CHO (30 n*M*). Activation of caspase-8, caspase-9, and caspase-3/7 as measured by Caspase-Glo assays 1 hour after TRAIL addition. Data show the mean activity ± SD for one representative experiment. **(C)** MTS assay to measure cell viability of different breast cancer cells after pretreatment with or without 5 μ*M* ABT-737 for 1 hour followed by treatment with 8 ng/ml (MB231, Hs578t) or 63 ng/ml (MB157, BT20, MB468, HCC1937, ZR75, T47D), 250 ng/ml (MB453, HCC1954), or 500 ng/ml (SKBR3) of TRAIL for 17 hours. Data shown are the mean ± SEM for three experiments for each cell line, and the data were compared by using a two-tailed Student's *t* test. *P < 0.05, comparing the combination treatment with TRAIL alone; **P < 0.05 comparing the combination treatment with ABT-737 alone; and ***P < 0.05 comparing the combination treatment with the sum of the inhibition by TRAIL alone plus the inhibitor alone.

Expression of BCL2L1 (BCL-XL) protein was measured in the four cell lines assayed in the secondary screen. BCL-XL was expressed in the four cell lines tested, but it was expressed at higher levels in the TRAIL-resistant T47D and SKBR3 cell lines (Additional file 9: Figure S5A). We confirmed the enhancement of TRAIL-induced caspase-3/7 activity by using five different *BCL2L1* siRNAs in the four cell lines used for in the secondary screen (siRNAs are listed in Additional file 2: Table S2). All five siRNAs enhanced TRAIL-induced caspase-3/7 activation by more than 2 standard deviations in the TNBC cell lines MB231 and MB468 and four of the five enhanced TRAIL-induced caspase-3/7 activation by more than 2 standard deviations in the ER-positive cell line T47D and in the HER2-amplified cell line SKBR3 (Figure 7A). These RNAi screens were performed in 384-well plates. To confirm that the enhancement of TRAIL activity correlated with the knockdown of BCL-XL protein, we tested two of the siRNAs (siBCL2L1.3 and siBCL2L1.5) in a larger-scale experiment on MB231 cells. In the previous plate experiments, knockdown of BCL-XL with siBCL2L1.3 consistently enhanced TRAIL-induced caspase-3/7 activity more than knockdown with siBCL2L1.5 (Figure 7A). In the larger-scale experiment, knockdown of BCL-XL by both siRNAs enhanced TRAIL-induced caspase-3/7 activity, and again, knockdown of BCL-XL with siBCL2L1.3 was more effective than knockdown with siBCL2L1.5 in enhancing TRAIL-induced caspase-3/7 activity across a wide range of TRAIL concentrations (see Additional file 9: Figure S5B).

Concordant with the effects on TRAIL-induced caspase-3/7 activation, siBCL2L1.3 resulted in a greater knockdown of the BCL-XL protein than siBCL2L1.5 (Additional file 9: Figure S5B). Thus the degree of BCL-XL protein knockdown correlated with the effect on TRAIL-mediated caspase-3/7 activity (Additional file 9: Figure S5B). Together, these data suggest that loss or inhibition of BCL2L1 may be useful in combination with TRAIL in a broad spectrum of breast cancer subtypes.

ABT-737 is an inhibitor of BCL-XL (BCL2L1), BCL-w (BCL2L2), and BCL-2 that has been shown to enhance cell death, including in MCF7 breast cancer cells and myeloma cells by binding and inhibiting the activity of antiapoptotic BCL2 family members [30,31]. Treatment of MB231 cells with ABT-737 resulted in increased TRAIL-induced activation of caspase-8, caspase-9, and caspase-3/7 (Figure 7B, compare striped bars with white bars). Unlike treatment of the cells with siRNA targeting BCL-XL, treatment of the MB231 cells with ABT-737 had no effect on the levels of BCL-XL protein (see Additional file 9: Figure S5C). Little or no increase was found in caspase-8, -9, or -3 activation when ABT-737 was added to cells in the absence of TRAIL (Figure 7B). Although an increase in caspase-9 and caspase-3 was expected by the inhibition of BCL2 family members by ABT-737, the increased TRAIL-induced activation of caspase-8 by ABT-737 was unexpected because ABT-737 works downstream of the initiator caspase-8. However, prior work demonstrated that caspase-8 can be activated by caspase-3 in a retrograde fashion, thus making it both an initiator and executioner caspase [21,22,32-35].

To test this, we measured the activation of caspase-8, -9, and -3/7 in the presence of the caspase-3/7 inhibitor DEVD-CHO. A low submaximal concentration of DEVD-CHO was used (30 n*M*), as this concentration was found to inhibit significantly TRAIL-induced caspase-3 activity but not to inhibit TRAIL-induced caspase-8 or caspase-9 activity directly (compare gray bars with white bars in Figure 7B). When cells were preincubated with the DEVD-CHO, no effect was seen on the TRAIL-induced activation of caspase-8 in the absence of ABT-737, but DEVD-CHO abrogated the ABT-737-induced increase in TRAIL-induced caspase-8 activation (Figure 7B, top panels). This is consistent with caspase-3/7 contributing to the increase in caspase-8 activation seen in the presence of ABT-737. Caspase-9 activation by TRAIL alone or by TRAIL plus ABT-737 was not affected by DEVD-CHO (Figure 7B, middle panels). DEVD-CHO significantly inhibited the TRAIL-induced activation of caspase-3 in both the presence and the absence of ABT-737 (Figure 7B, lower panels). These data are consistent with ABT-737 causing increased caspase-9 activation by caspase-8. This, in turn, results in more caspase-3/7 activation and then retrograde activation of caspase-8 by caspase-3/7.

Treatment of a panel of breast cancer cell lines with 5 μ*M* ABT-737 (Figure 7C) by using sub-IC_50_ concentrations of TRAIL, enhanced TRAIL-induced toxicity in all of the breast cancer subtypes tested (TNBC/Basal B, TNBC/Basal A, HER2 amplified, and ER + breast cancer) (Figure 7C). The combined treatment of TRAIL plus ABT-737 inhibited viability more than TRAIL alone (*P < 0.05) or ABT-737 alone (**P ≤ 0.05) in all cells tested. The toxicity of the combined treatment was greater than the sum of the toxicities for the individual treatments for all cell lines (***P ≤ 0.05), except for MB157. Again the high sensitivity to TRAIL alone in this cell line probably accounts for the failure of ABT-737 to enhance significantly the toxicity by this analysis.

## Discussion

TRAIL is a promising cancer therapeutic agent showing efficacy against tumor cells and not affecting normal cells. However, *in vitro* experiments have found that many cancer cell lines are resistant to TRAIL [5]. The underlying determinants of TRAIL sensitivity are not clearly understood. Investigations into the mechanisms in cells that regulate sensitivity to TRAIL have implicated several pathways and factors. Regulation of the TRAIL receptors at the level of expression, localization to the cell surface, and *O*-glycosylation of the receptor proteins partially, but not fully, correlate with sensitivity (reviewed in [5]). TRAIL-resistance is also associated with elevated expression of antiapoptotic factors like c-FLIP [36], IAP family proteins [37], and BCL-2 [38].

In ongoing clinical trials, responses to TRAIL have been rare, especially in solid tumors [21,22,32-35]. Therefore we need to identify proteins that regulate the TRAIL pathway, as they could potentially serve as predictive biomarkers of TRAIL sensitivity and/or provide additional targets for enhancing the efficacy of TRAIL.

To this end, we performed primary siRNA screens of the human kinome, phosphatome, and some additional genes to identify regulators of TRAIL-induced apoptosis in the MB231 breast cancer cell line. We identified 150 genes (83 kinases or kinase-related genes, four phosphatases or phosphatase-related genes, and 63 other genes) as putative negative regulators of TRAIL-induced caspase-3/7 activation. For this study, we adapted commercially available assays of caspase-8, caspase-3/7, and cell viability for high-throughput siRNA screens, including the identification of highly sensitive biologically relevant controls. Good positive correlation was found between those siRNAs that enhanced TRAIL-induced caspase-3/7 and those that enhanced TRAIL-induced caspase-8 activation (Figure 2Ci-ii). Good inverse correlation was seen between the TRAIL-induced enhancement of caspase activation and the viability of TRAIL-treated cells. Thus, the three assays together strengthen the likelihood that the identified genes are regulators of the TRAIL pathway. The identification of several established negative regulators of apoptosis as negative regulators of TRAIL-induced caspase-3/7 activation, including *BCL2L1* (BCL-XL), *BCL2L2* (BCL-w), *BIRC2* (c-IAP1), and *BIRC3* (c-IAP2), lends further support to the validity of the screen results.

Interestingly, other candidate genes identified by our screens have been linked recently to TRAIL activity. For example, the expression of argininosuccinate synthase 1 (*ASS1*) has been described as a member of a predictive panel of 71 genes whose expression correlates with TRAIL sensitivity [39]. *ASS1* was the only gene in common between the 71-gene signature and the set of genes found in our screen. Based on our experiments, *ASS1* is a putative negative regulator of TRAIL sensitivity, and LOF induced an increase in caspase-3/7 activation (Figure 3). *ASS1* is the rate-limiting enzyme in arginine biosynthesis, and interestingly, two studies demonstrated that loss of *ASS1* sensitizes lymphoma and glioblastoma cells to apoptosis induced by arginine deprivation [40,41]. The LOF of *ASS1*, then, may result in arginine depletion and make cells more susceptible to TRAIL-induced apoptosis. Elucidating the mechanism by which *ASS1* negatively regulates TRAIL-induced apoptosis will require further study.

Among the approximately 1,300 genes assessed at the higher stringency (that of a 2-standard deviation change in TRAIL-induced caspase-3/7 activity), these RNAi screens did not identify positive regulators of TRAIL. Several potential positive TRAIL regulators were identified when the stringency was relaxed to a 1-SD change in TRAIL-induced caspase-3/7 activation (Additional file 3: Figure S1). None of these putative positive regulators has been linked previously to the regulation of TRAIL-induced apoptosis or apoptosis in general, although one of the genes identified, *PXK*, has been recently shown to enhance degradation of the activated epidermal growth factor receptor (EGFR) [42]. We and others have shown that EGFR activity can attenuate TRAIL-induced apoptosis and that inhibition of the EGFR enhances TRAIL-induced apoptosis [20,43,44]. Thus, PXK LOF may enhance TRAIL activity by the downregulation of the EGFR, although this hypothesis will require further study.

The absence of strong positive regulators in our RNAi screens suggests that the primary regulation of TRAIL-induced apoptosis is by inhibition of the TRAIL pathway. However, our screen was focused on kinases and phosphatases and included only 300 additional genes from the druggable genome. Notably, caspase genes were not among the screened targets, although, as shown by the caspase-8 controls in our screen, these would have been identified as positive regulators of TRAIL-induced caspase activation and apoptosis. To identify positive regulators of TRAIL-induced apoptosis, more-comprehensive, genome-wide RNAi screens, using the assays developed for this study, are quite likely to identify other positive regulators (and negative regulators) of TRAIL.

A previous RNAi-based screen of 510 genes conducted in HeLa cells identified both positive and negative regulators of the TRAIL pathway [45]. The reported screen included many kinases as candidate regulators of TRAIL, but little overlap existed between our results and the results reported by Aza-Blanc and co-workers. Of the top 20 negative TRAIL-regulator genes identified in the previous study, only *PIP5K1C* was identified in our screens, and none of the top 20 positive TRAIL-regulator genes described in the previous report was among the positive regulators our screen identified at relaxed stringency.

The differing results are likely the result of several significant differences in our screens. First, we performed the screen in a TNBC breast cancer cell line, whereas the prior study was performed in the cervical carcinoma HeLa cell line. It is likely that the predominant regulators of TRAIL-induced apoptosis are different in different cell types. Second, our primary selection of genes whose LOF altered TRAIL activity was based on caspase-3/7 activation 1 hour after the addition of TRAIL, whereas the previous study measured viability 20 hours after the addition of TRAIL. Thus, our screens were designed principally to identify regulators that affect early steps in TRAIL-induced apoptosis, contributing to the difference noted.

Review of the putative negative regulators identified in our primary RNAi screens in MB231 revealed genes involved in diverse cellular processes, including growth factor receptor signaling (for example, *BTK*, *ERBB-2*, *EPH6*, *ERK8*, *FGFR2*, *FGFR4*, *JAK1*, and *SRC*), cytoskeleton function (for example,, *ACTN4*, *KIF1A*, *LIMK2*, *MAGI1*, and *PKN1*), bioenergetics (for example, *ACACB*, *ACLY*, *ATP5A1*, *CKB*, *CKMT2*, *FN3K*, *HK1*, *HK2*, *IHPK3*, *PDK2*, *PFKL*, and *PKLR*), cell-cycle regulation (for example, *CDK5R2*, *CDKN2B*, *GAK*, *PIK3*, *PFTK1*, and *ZC3HC*1), transcriptional regulation (for example, *HIPK1*, *HIPK2*, *NLK*, and *PIM2*), and DNA repair (for example, *APEX1*, *PARP4*, and *TLK*). Also of note, several genes known to regulate apoptosis negatively were identified (for example, *AATK*, *BCL2L1*, *BCL2L2*, *BIRC2*, *BIRC3*, *IKBKAP*, *IKBKB*, *PRKC*I, *PIM2*, and *SPHK2*).

The largest gene set in our RNAi library included the known kinases and kinase-associated genes. Of the group of kinases that were identified as hits, the majority of them are serine/threonine kinases (33 of 83), whereas fewer belonged to the tyrosine kinase (10 of 83), lipid kinase (four of 83), or sugar/metabolism kinase (12 of 83) families. Interestingly, four kinases were identified (hexokinase 1 (HK1), hexokinase 2 (HK2), pyruvate kinase liver and red blood cells (PKLR), and phosphofructose kinase liver (PKFL)), which regulate irreversible steps of the glycolysis pathway (Figure 3). Several studies have previously found that inhibition of glycolysis enhances TRAIL-induced cell death [46-49].

Based on the gene-network analysis, four genes were identified that appear at central nodes of an interaction map generated by using the caspase-3/7 screening dataset, *PDPK1*, *IKBKB*, *SRC*, and *BCL2L1* (BCL-XL) (Figure 4A). The caspase-8 and cell-viability screening data confirmed these findings for *BCL2L1* (BCL-XL) and *PDPK1*. PDPK1 phosphorylates and activates AKT. Constitutively active or overexpression of AKT has been shown to confer TRAIL resistance in several tumor types, including breast [18], lung [50], gastric [51], and prostate [52]. Also, TRAIL can activate SRC, leading to AKT activation and TRAIL resistance [29]. Inhibition of the PI3 kinase/AKT pathway has been found to enhance TRAIL-induced apoptosis [43,50,51,53-57]. Therefore, identification of PDPK1 as one of the key nodes provides a rationale for pursuing studies on the combination of TRAIL with AKT inhibitors in treatment of TRAIL-resistant tumors.

NF-κB proteins are ubiquitously expressed proteins that can protect cells from apoptosis. The inhibitors of κB (IκB) family proteins regulate the activity of NF-κB. IκB proteins block nuclear localization signals of functional NF-κB dimers by binding to dimerization domains and sequestering the dimers in the cytoplasm. IκB kinases (IκBK) phosphorylate IκB on a serine residue, targeting them for proteasomal degradation, thereby activating NF-κB, which protects cells by increasing the expression of antiapoptotic proteins [3,58,59]. Previously, we showed that inhibition of NF-κB increases TRAIL sensitivity in breast cancer cell lines [18]. Similar results were reported in other cancer cell lines [18,60-63]. Again, our findings in this article that IκBKB LOF leads to enhanced TRAIL-induced caspase activation provide support for further studies of NF-κB inhibitors in combination with TRAIL.

Further to confirm our primary screen results, we performed a secondary screen of 16 genes identified as negative regulators of TRAIL-induced caspase activation in four cell lines representing different subtypes of breast cancer (TNBC, ER positive, and HER2 amplified) (Figure 5, Table 1, and Additional file 8: Table S4). We selected 16 genes that were included in the network analysis in Figure 4 and that both increased TRAIL-induced caspase-3/7 activity and enhanced TRAIL-induced toxicity in a viability assay. In MB231, 13 of 16 genes scored positive in this assay (by using the criterion of 2-SD enhancement in TRAIL-induced caspase-3/7 activation), and 15 of 16 genes scored positive at a lower-stringency cutoff (by using the criterion of 1-SD enhancement in TRAIL-induced caspase-3/7 activation). This high level of reproducibility between the primary and secondary screen in MB231 supports the validity of the primary screen. All of the 16 genes scored positive by using the high-stringency criterion in MB468. The TNBC/basal A MB468 cell line is most closely related to the TNBC/basal B MB231 cell line by cDNA microarray expression analysis, and thus the high degree of overlap between the two cell lines in this screen is not surprising [26,64]. By contrast, fewer of the 16 genes were scored positive in T47D (four at high stringency and 10 at low stringency) and SKBR3 (two at high stringency and three at low stringency). The T47D cell line is an ER-positive luminal breast cancer cell line, and the SKBR3 cell line is an HER2-amplified luminal breast cancer cell line. Thus they are more distantly related to the MB231 cell line [26,64].

The only gene that scored positive in our screen at high stringency in all four cell lines is alpha-actinin 4 (*ACTN4*). ACTN4 is a cytoskeletal protein that has been found to interact with signaling molecules, chromatin-remodeling factors, and transcription factors (reviewed in [65]). Of note, ACTN4 can serve as a scaffold to promote AKT activation, and it has been shown to interact with NF-κB in breast cancer cells (although the significance of this latter interaction is not known) [65]. Thus, it is plausible that by modulating activity through these two antiapoptotic pathways, ACTN4 might serve as a negative regulator of TRAIL-induced apoptosis. The mechanisms by which ACTN4 regulate TRAIL-induced apoptosis in breast cancer cells will require further investigation. LOF of *BCL2L1* (BCL-XL) enhanced TRAIL-induced caspase-3/7 activation in three of the four cell lines at high stringency and in all four cell lines when a lower stringency was used. Expanded screening of five *BCL2L1* siRNAs confirmed that BCL2L1 LOF results in enhanced TRAIL activity in four breast cancer cell lines (Figure 7A). A number of studies have directly or indirectly implicated the BCL2 family as regulators of TRAIL-induced apoptosis in breast cancer cells [33,66-71].

In this study, we identified *BCL2L1* (BCL-XL) as a key node in determining sensitivity (Figure 4) and further showed that inhibition of the BCL-2 family by the small-molecule inhibitor, ABT-737, enhances TRAIL-induced toxicity in breast cancer cell lines (Figure 7C). These results are in concordance with previous reports of the combined use of TRAIL and ABT-737 in renal, lung, prostate, and pancreatic cancer cell lines [72,73]. ABT-737 is a BH3 mimetic inhibitor of BCL-XL, BCL-2, and BCL-w [74]. Interestingly, both BCL-XL (*BCL2L1*) and BCL-w (*BCL2L2*) were identified as negative regulators of TRAIL-induced caspase-3/7 activation in the breast cancer cells by our primary screen. This suggests that the effects of ABT-737 may be due to inhibition of multiple BCL2 family members. Most important, the concomitant treatment with ABT-737 and TRAIL resulted in significantly more cell death in both sensitive and resistant breast cancer cell lines of all phenotypes (Figure 7C). This suggests that the BCL2 family may play a role more broadly in regulating TRAIL sensitivity in breast cancer cells and is worth further investigation.

SRC enhanced TRAIL-induced caspase-3/7 activation in the two TNBC cell lines at high stringency (MB231 and MB468) and in the T47D cell line at lower stringency. SRC is an important kinase regulating cell-survival pathways [75]. In our study, inhibition of SRC resulted in a decrease in the activity of the PI3K/AKT/mTOR pathway, consistent with published findings that SRC regulates the activity of the PI3K/AKT/mTOR and that inhibition of this pathway increases TRAIL sensitivity (Figure 6B) [28,29,76-79]. In the present study we showed that SRC is a key node of TRAIL-induced apoptosis, as illustrated in the pathway-analysis map (Figure 4A), and that inhibition of SRC by PP2 increases the sensitivity of breast cancer cells to TRAIL (Figure 6) [28,29]. The most significant effects of SRC inhibition on TRAIL-induced cell death were observed in the TNBC cells (both basal A and basal B). The TNBC/basal A breast cancer cell lines are relatively resistant to TRAIL compared with the TNBC/basal B cell lines [20]. Our data raise the possibility that combinations of TRAIL and SRC inhibitors may be of use in TNBC. The effects of TRAIL plus PP2 in the HER2-amplified and ER-positive cells were less dramatic. Although the reason for this is not clear, the focus of further studies with SRC inhibitors combined with TRAIL should be in TNBC cells.

## Conclusions

In this study, we successfully applied complementary siRNA screens by using different end-point assays to identify negative regulators of TRAIL-induced apoptosis in breast cancer cells. The identification of *PDPK1*, *IKBKB*, *SRC,* and *BCL2L1* as central nodes connecting the genes identified is consistent with previous studies. Importantly, this study demonstrates that phenocopying *SRC* and *BCL2L1* LOF by pharmacologic inhibition can sensitize TRAIL-resistant breast cancer cell lines to TRAIL-induced apoptosis. In these screens, we identified a large number of additional genes as potential regulators of TRAIL-induced apoptosis, whose role in the TRAIL pathway is heretofore unknown. It will require further study to elucidate how they regulate the TRAIL pathway. The genes identified by this screen are likely to include novel therapeutic targets that can be tested in combination with TRAIL in treating a variety of tumors, including breast cancer.

## Abbreviations

Casp: caspase; DISC: death-inducing signaling complex; FBS: fetal bovine serum; FLIP: FLICE-like inhibitory protein; RNAi: RNA interference; siRNA: small interfering RNA; TNBC: - triple-negative breast cancer; TRAIL: tumor necrosis-related apoptosis-inducing ligand.

## Competing interests

The authors declare that they have no competing interests.

## Authors’ contributions

All authors made substantial contributions to conception, design, implementation, analysis, and presentation of this study. NJC and SL are co-corresponding authors of this work. SVG, NJC, and SL conceived of and designed the overall experimental approaches. SVG, JLD, SC, KG, and MG designed, carried out, and analyzed the experiments. JJP and NJC conceived of, carried out, and interpreted the bioinformatics analyses. MMN contributed additional data analysis and interpretation of the results. All authors reviewed the data and contributed to the writing of the manuscript. All authors read and approved the final manuscript.

## Supplementary Material

Additional file 1: Table S1 Caspase-8, caspase-3/7, and cell viability siRNA primary screening data in MB-231 cells in the absence and presence of TRAIL. Data are for four different siRNAs per gene (A, B, C, and D), shown as fold-change relative to siNeg-transfected cells in the absence of TRAIL. Click here for file

Additional file 2: Table S2 Genes and siRNA sequences selected for secondary screening of putative regulators of TRAIL-induced apoptosis. Click here for file

Additional file 3: Figure S1 Caspase-3/7 and cell-viability RNAi screens of the phosphatome and TRAIL-induced apoptosis in MB231 cells. **(A)** Controls included in the RNAi screens of the phosphatome gene set in MB231 cells for (i) caspase-3/7 activation and (ii) cell viability in the absence (white bars) or presence (black bars) of TRAIL. Cells were siRNA-transfected, treated with TRAIL, and assessed for caspase-3/7 activation and cell viability, as described in Figure 2. Data are normalized to the mean value of siNeg-transfected cells in the absence of TRAIL and are shown as the mean and standard deviation for each group. Comparison of TRAIL treated with siNeg-transfected untreated cells demonstrated a significant increase in caspase-3/7 activation and a significant decrease in viability. siCASP8 reduced caspase-3/7 activation (*P* = 1.0 × 10^-14^) and increased viability (*P* = 1.5 × 10^-14^) compared with siNeg-transfected cells. siFLIP increased caspase-3/7 (*P* = 1 × 10^-8^) activation and decreased viability (*P* = 7.0 × 10^-7^) compared with siNeg-transfected cells. ***P < 0.001. **(B)** To assess further the sensitivity of our assays, we confirmed that silencing Polo-like kinase 1 (PLK1), an essential protein in many cell lines, activated caspase-8, caspase-3/7, and decreased cell viability. The dashed line indicates the 1-SD value for each assay; the red dots indicate those siRNAs inducing fold-changes greater than 1 SD. **(C)** Identification of putative positive regulators of TRAIL-induced apoptosis. Genes for which three or four siRNAs decreased activation of caspase-3/7 in the presence of TRAIL 1 or more SDs over that observed in siNeg-transfected cells plus TRAIL were considered as putative positive regulators of TRAIL-induced apoptosis. The dashed line indicates the 1-SD fold-change, and a vertical line joins those siRNAs that induced at least this level of change for each gene. Data for the control siRNAs, siNeg, siCASP8, and siFLIP are included for reference. Click here for file

Additional file 4: Table S3 Primary screen Z-factors calculated for the viability, caspase-3/7, and caspase-8 assay plates. Click here for file

Additional file 5: Figure S2 Interaction network analysis of putative negative regulators of TRAIL-induced apoptosis. An interaction network generated by analysis of the 150 genes for which three or more siRNAs induced increased TRAIL-induced activation of caspase-3/7 levels. All symbols are presented as depicted by the Ingenuity Pathway Analysis software. Gene names in black and linked by solid lines indicate evidence for a mechanistic relation between the proteins indicated. Gene names in blue and linked by dashed lines indicate correlative relations between the proteins indicated, but no mechanistic relation has been established. Click here for file

Additional file 6: Figure S3 Validation of genes identified by interaction analysis. Caspase-8 and caspase-3/7 activation, and cell viability in the absence (empty bars) and presence (black bars) of TRAIL for four siRNAs **(A-D)** corresponding to the genes shown. Mean data (±1 standard deviation) for control siRNA (siNeg) transfected cells are shown in each graph. The dashed line indicates the relevant 1 SD value for each assay, and the red dots indicate those siRNAs inducing fold-changes greater than 1 SD. Click here for file

Additional file 7: Figure S4 Caspase-3/7 activation by TRAIL in breast cancer cell lines. Cell lines were treated with increasing concentrations of TRAIL, as indicated along the X axis for 1 hour, and caspase-3/7 activation was measured by Caspase-Glo-3/7 assay, as described earlier. Click here for file

Additional file 8: Table S4 Caspase-3/7 siRNA secondary screen in a panel of breast cancer cell lines in the absence and presence of TRAIL. Data are for four different siRNAs per gene, shown as fold-change relative to siNeg-transfected cells in the absence of TRAIL. Values indicated in red are >2 SDs higher than TRAIL-induced caspase-3/7 in siNeg-treated cells. Values indicated in blue are >1 SD higher than TRAIL-induced caspase-3/7 in control siRNA (siNeg)-treated cells. Click here for file

Additional file 9: Figure S5 BCL-XL protein expression in the breast cancer cells. **(A)** Expression of BCL-XL was measured by immunoblotting in the four cell lines used in the secondary RNAi screen. **(B)** Two BCL-XL-specific siRNAs (*siBCL2L1.3* and *siBCL2L1.5*) were compared with a negative control siRNA (siNEG) for their ability to enhance TRAIL-induced caspase-3/7 activity, as described earlier and in parallel for their knockdown of BCL-XL protein. **(C)** Levels of BCL-XL protein were measured by immunoblotting in cells treated with ABT-737 (5 μ*M*) for the times indicated. In all of the blots, HSC70 is shown as a loading control, and MW in kilodaltons is shown to the left of the panels. Click here for file
