# Supplementary material for: Identification of novel molecular regulators of tumor necrosis factor-related apoptosis-inducing ligand (TRAIL)-induced apoptosis in breast cancer cells by RNAi screening
Source: Breast Cancer Res. 2014 Apr 17;16(2):R41. doi: 10.1186/bcr3645 (PMC4053258; doi:10.1186/bcr3645)
Supplement: Additional file 1: Table S1 — Caspase-8, caspase-3/7, and cell viability siRNA primary screening data in MB-231 cells in the absence and presence of TRAIL. Data are for four different siRNAs per gene (A, B, C, and D), shown as fold-change relative to siNeg-transfected cells in the absence of TRAIL. [file bcr3645-S1.pdf]

NCBI gene symbol	Entrez Gene Id	Gene Description	mRNA Accessions	siRNA Target Sequence 5'-3'	Product Id (Qiagen (Inc.)	Vendor validated	In primary screen	siRNA Name
ACTN4	81	actinin, alpha 4	NM_004924	CCCGCAAATCATCAACTCCAA	SI02779980	Yes	Yes	siACTN4.1
				ACGCAGCATCGTGGACTACAA	SI02779973	Yes	Yes	siACTN4.2
				AAGGTGCTGGCTGTCAACCAA	SI00070098		Yes	siACTN4.3
				CTGACACATAGTCGCAGGGAA	SI00070084		Yes	siACTN4.4
ATP5A1	498	ATP synthase, H+ transporting, mitochondrial F1 complex, alpha subunit 1, cardiac muscle	NM_001001935 NM_001001937 NM_004046	TTGGCTGGATTTGAAGCTTAA	SI02776991	Yes		siATP5A1.1
				ATGCTTTGGGTTCATCTTTCA	SI00306859		Yes	siATP5A1.2
				CCCAGTTGGTGAAGAGACTTA	SI00306852		Yes	siATP5A1.3
				CCCGGTATCATTCCTCGAATT	SI00306866		Yes	siATP5A1.4
BCL2L1	598	BCL2-like 1	NM_001191 NM_138578	CAGCTGCCTCACTTCCTACAA	SI03068352			siBCL2L1.1
				TTGGCTTTGGATCTTAGAAGA	SI03025141			siBCL2L1.2
				TAGGGTGGCCCTTGCAGTTCA	SI03112018			siBCL2L1.3
				CTGCTTGGGATAAAGATGCAA	SI00023191			siBCL2L1.4
				AAGGCGGATTTGAATCTCTTT	Custom		Yes	siBCL2L1.5
BCR	613	breakpoint cluster region	NM_004327 NM_021574	CAGCCGCAACGGCAAGAGTTA	SI00062762		Yes	siBCR.1
				CAGCATTCCGCTGACCATCAA	SI00288141	Yes	Yes	siBCR.2
				ACGGCAGTCCATGACGGTGAA	SI00288134	Yes	Yes	siBCR.3
				AAGGTCAACGACAAAGAGGTG	SI00299425	Yes		siBCR.4
BIRC2	329	baculoviral IAP repeat containing 2	NM_001166	AACATAGTAGCTTGTTCAGTG	SI02654435	Yes		siBIRC2.1
				CTAGGAGACAGTCCTATTCAA	SI02654442	Yes		siBIRC2.2
				TAGGCGTTTCTGATAACACTA	SI00022855		Yes	siBIRC2.3
				TCCCAGGTCCCTCGTATCAAA	SI02625427		Yes	siBIRC2.4
CNKSR1	10256	connector enhancer of kinase suppressor of Ras 1	NM_006314	CAGGGTGGCGTGTCCCTCCTA	SI03571169	Yes		siCNKSR1.1
				CAGGAGCTGCAGGTCCTAGAA	SI00087402		Yes	siCNKSR1.2
				GAGGGCTACTTTCCAACCAAA	SI00087395		Yes	siCNKSR1.3
				ACCCATGACTTCCAGAGCATA	SI02665411	Yes		siCNKSR1.4
FGFR4	2264	fibroblast growth factor receptor 4	NM_002011 NM_022963 NM_213647	CAGGCTCTTCCGGCAAGTCAA	SI02665306	Yes		siFGFR4.1
				CAGGAGGTTCTGGGCCTCTGA	SI00031374		Yes	siFGFR4.2
				CCGCCTGACCTTCGGACCCTA	SI02659979	Yes	Yes	siFGFR4.3
				CACATTGACTACTATAAGAAA	SI00031360		Yes	siFGFR4.4
HIPK2	28996	homeodomain interacting protein kinase 2	NM_001113239 NM_022740	AAGCGTCGGGTGAATATGTAT	SI04439393			siHIPK2.1
				TCCCGAAGTCTCCATACTAAA	SI02659818	Yes		siHIPK2.2
				CCAGGTGAACATGACGACAGA	SI04439386			siHIPK2.3
				AACCAGTACCCTTACATATAA	SI02659811	Yes		siHIPK2.4
IKBKB	3551	inhibitor of kappa light polypeptide gene enhancer in B-cells, kinase beta	NM_001190720 NM_001190721 NM_001242778 NM_001556	TAGGGAGCTATTCATGTTTCA	SI02626456		Yes	siIKBKB.1
				CAGACCGACATTGTGGACTTA	SI02626442		Yes	siIKBKB.2
				CTGGAGAAGTACAGCGAGCAA	SI02777376	Yes		siIKBKB.3
				AAACCGAGTTTGGGATCACAT	SI00300545	Yes		siIKKB2.4
MKNK1	8569	MAP kinase interacting serine/threonine kinase 1	NM_001135553 NM_003684 NM_198973	TAGATAGTGCTCTGTGCCTAA	SI02621962	Yes		siMKNK1.1
				CACGGACCAGGCCACATTCTA	SI00054663			siMKNK1.2
				CAGCACGAAGAGAACGAACTA	SI00054649		Yes	siMKNK1.3
				CCAGCAAAGATGATACCTTAA	SI02621955	Yes	Yes	siMKNK1.4
PDPK1	5170	3-phosphoinositide dependent protein kinase-1	NM_002613 NM_031268	AACAAAGTTCTGAAAGGTGAA	SI00301154	Yes		siPDPK1.1
				CACGCCTAACAGGACGTATTA	SI00605787	Yes	Yes	siPDPK1.2
				AAGGGCATCATTCACAGGGAC	SI00301140	Yes		siPDPK1.3
				AAGCGGTTAGGCTGTGAGGAA	SI00605780	Yes	Yes	siPDPK1.4
PIP5K1C	23396	phosphatidylinositol-4-phosphate 5-kinase, type I, gamma	NM_001195733 NM_012398	CGCGCCCGCCACCGACATCTA	SI02758966	Yes		siPIP5K1C.1
				CCGCGTCGTGGTCATGAACAA	SI02224376	Yes	Yes	siPIP5K1C.2
				CGGCGAAACCACCTACAAGAA	SI00099967		Yes	siPIP5K1C.3
				CGGCAAGACCTATTTATAATA	SI00099953		Yes	siPIP5K1C.4
PLK3	1263	polo-like kinase 3	NM_004073	CCCGAAATCGTAGTGCTTGTA	SI02223466	Yes	Yes	siPLK3.1
				GTGGGTTGACTACTCCAATAA	SI00059395		Yes	siPLK3.2
				CAGAAAGACTGTGCACTACAA	SI00059388		Yes	siPLK3.3
				CTGCATCAAGCAGGTTCACTA	SI02223473	Yes	Yes	siPLK3.4
PRKCI	5584	protein kinase C, iota	NM_002740	AACAGGTGGTACCTCCCTTTA	SI00301336	Yes		siPRKCI.1
				ACGCCGCTGGAGAAAGCTTTA	SI02660084	Yes	Yes	siPRKCI.2
				CAGATTGTTCTTTGTTATAGA	SI02660098	Yes		siPRKCI.3
				TCCGGGTAATAGGAAGAGGAA	SI02660091	Yes	Yes	siPRKCI.4
RIOK3	8780	RIO kinase 3 (yeast)	NM_003831 NM_145906	CAGACTGTGGCCGTGATTCTA	SI00056525		Yes	siRIOK3.1
				ATGGGTGACTTGAAATACCAA	SI00056504		Yes	siRIOK3.2
				CTGGCTGTATTAGTACAGGAA	SI02223403	Yes	Yes	siRIOK3.3
				ATGCGGCAGTTATATCATGAA	SI02223396	Yes	Yes	siRIOK3.4
SRC	6714	v-src sarcoma (Schmidt-Ruppin A-2) viral oncogene homolog (avian)	NM_005417 NM_198291	CTCCATGTGCGTCCATATTTA	SI02664151	Yes	Yes	siSRC.1
				TCCATGTGCGTCCATATTTAA	SI00076832		Yes	siSRC.2
				AAGCAGTGCCTGCCTATGAAA	SI02223921	Yes	Yes	siSRC.3
				CGGCTTGTGGGTGATGTTTGA	SI02223928	Yes	Yes	siSRC.4
