# Supplementary figures and images for: Identification of novel molecular regulators of tumor necrosis factor-related apoptosis-inducing ligand (TRAIL)-induced apoptosis in breast cancer cells by RNAi screening
Source: Breast Cancer Res. 2014 Apr 17;16(2):R41. doi: 10.1186/bcr3645 (PMC4053258; doi:10.1186/bcr3645)

A

Figure S1

i

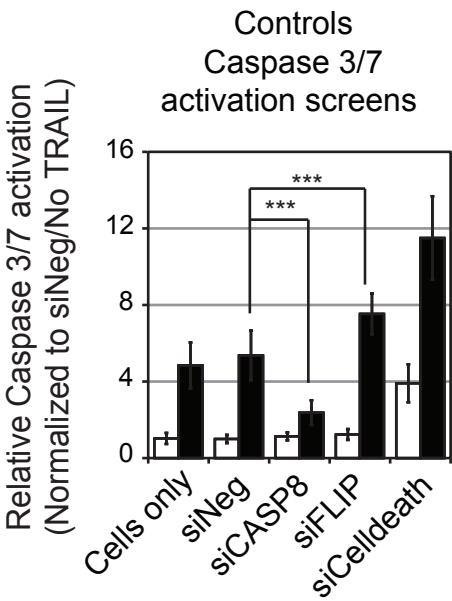

ii

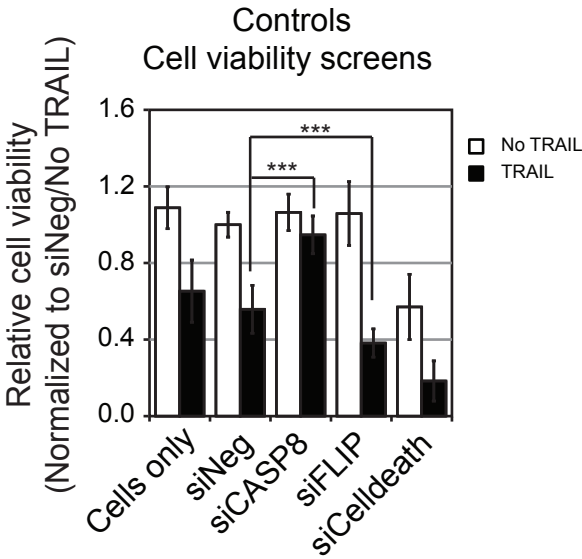

B

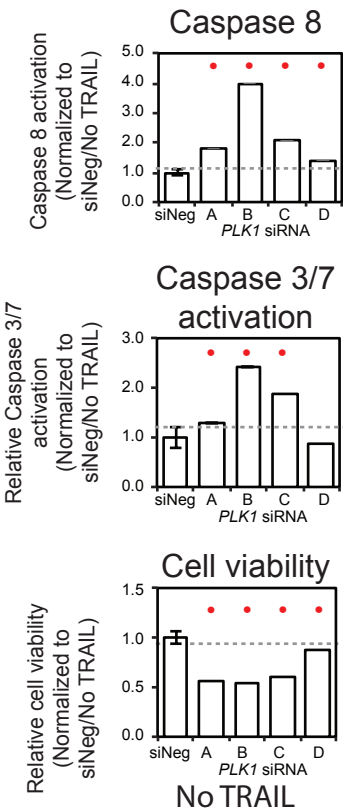

C

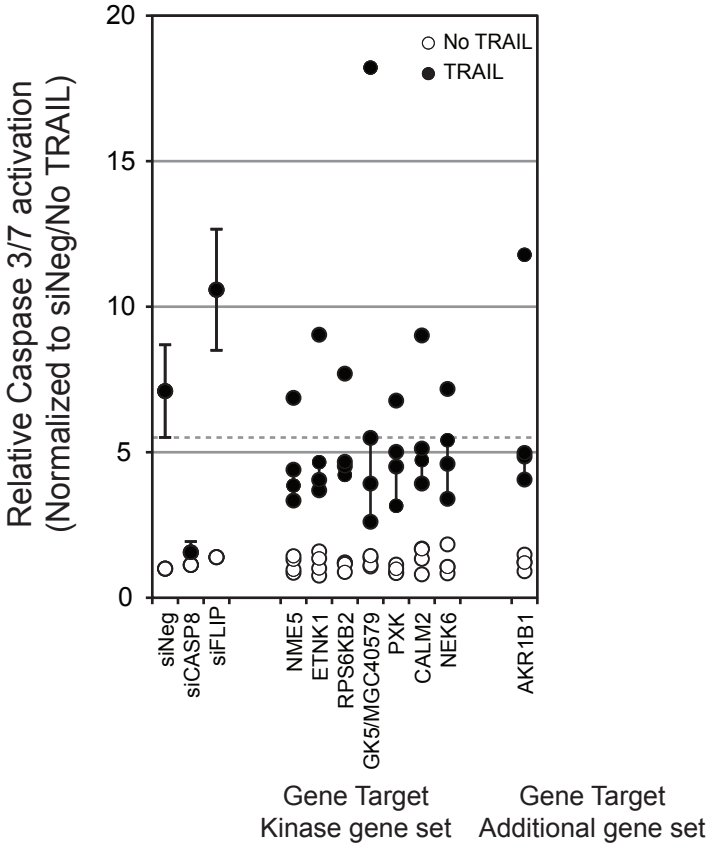

Supplement: Additional file 2: Table S2 — Genes and siRNA sequences selected for secondary screening of putative regulators of TRAIL-induced apoptosis. [file bcr3645-S2.pdf]

### Figure S2

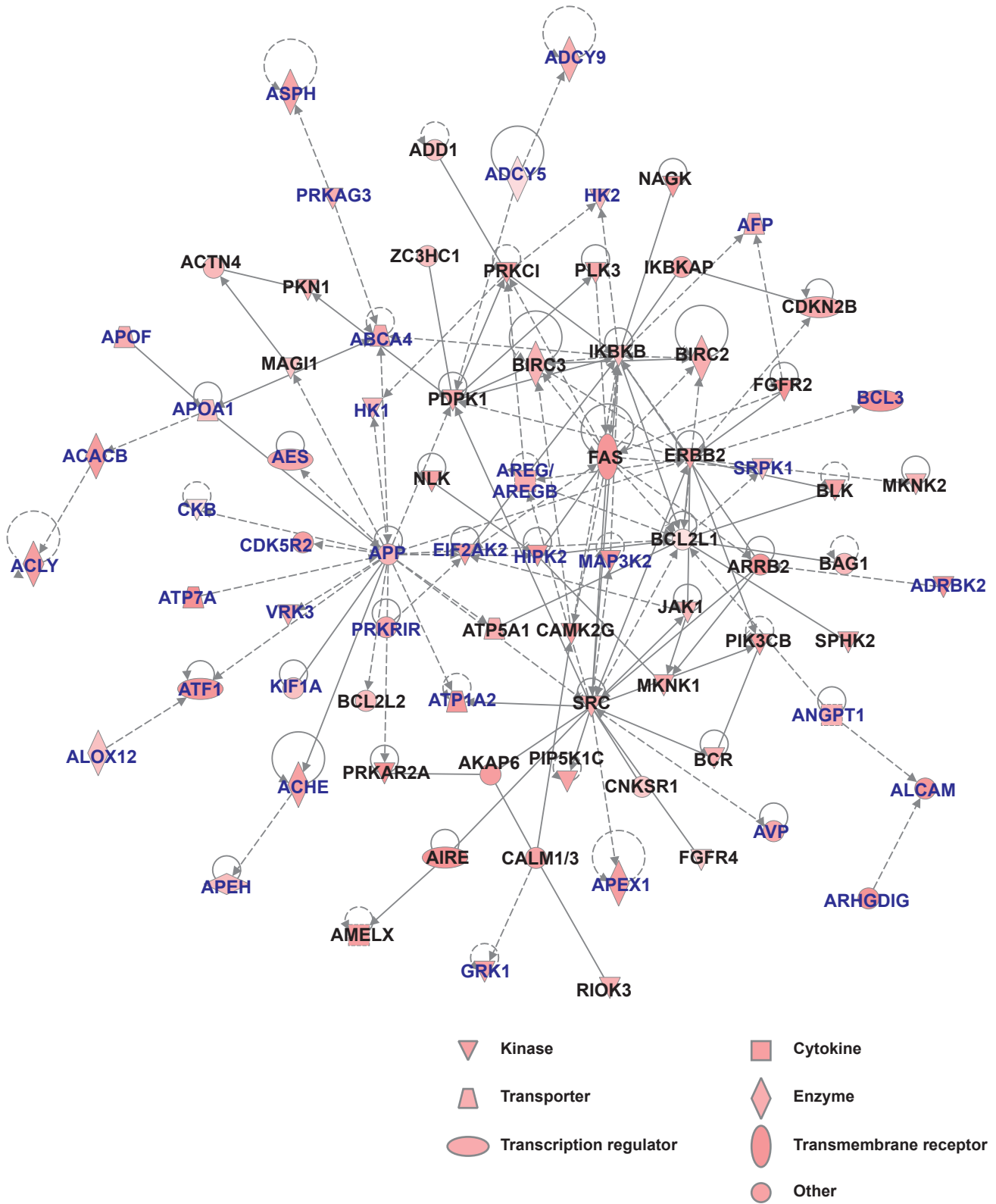

Supplement: Additional file 4: Table S3 — Primary screen Z-factors calculated for the viability, caspase-3/7, and caspase-8 assay plates. [file bcr3645-S4.pdf]

**Figure S3**

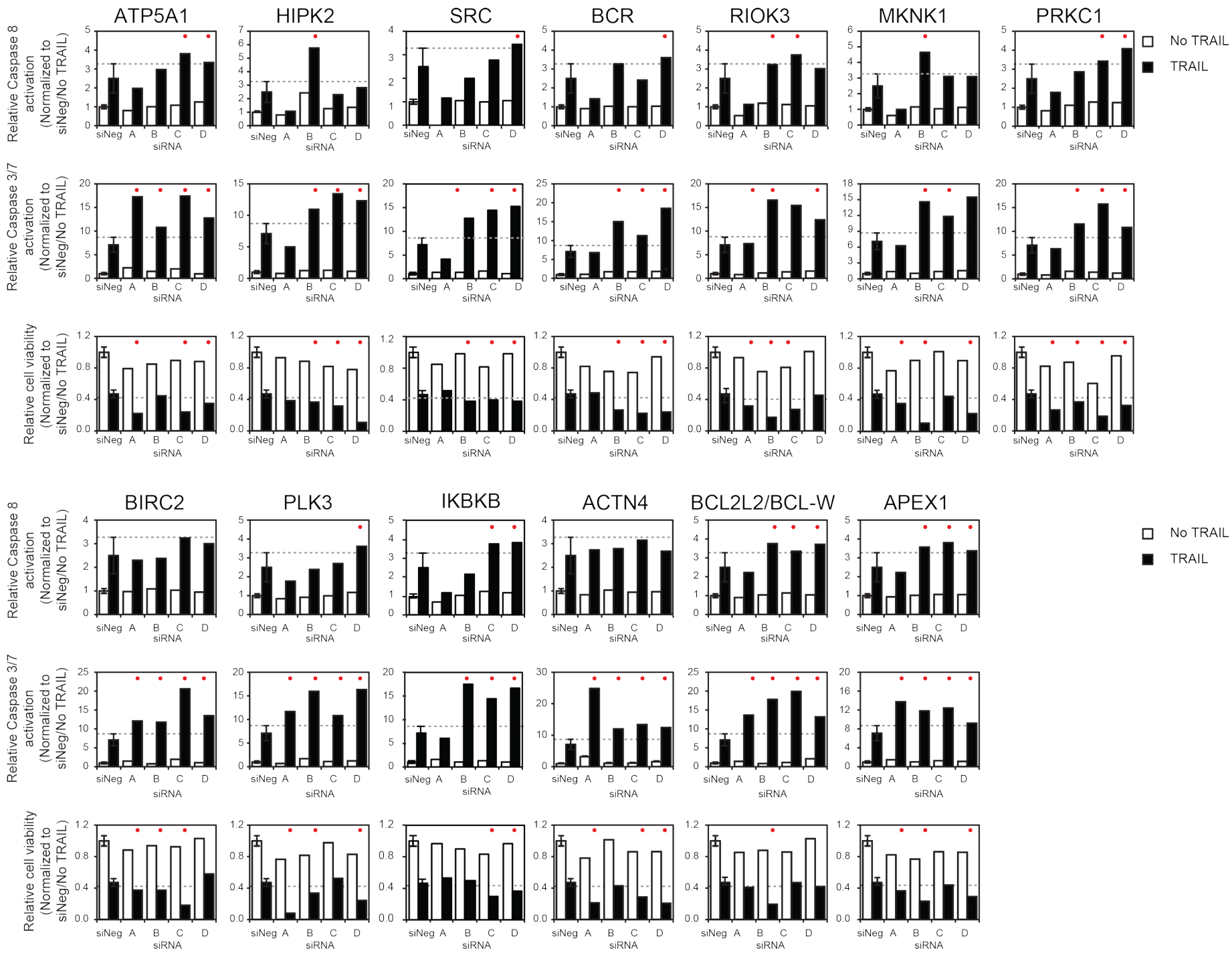

Supplement: Additional file 5: Figure S2 — Interaction network analysis of putative negative regulators of TRAIL-induced apoptosis. An interaction network generated by analysis of the 150 genes for which three or more siRNAs induced increased TRAIL-induced activation of caspase-3/7 levels. All symbols are presented as depicted by the Ingenuity Pathway Analysis software. Gene names in black and linked by solid lines indicate evidence for a mechanistic relation between the proteins indicated. Gene names in blue and linked by dashed lines indicate correlative relations between the proteins indicated, but no mechanistic relation has been established. [file bcr3645-S5.pdf]

Figure S4

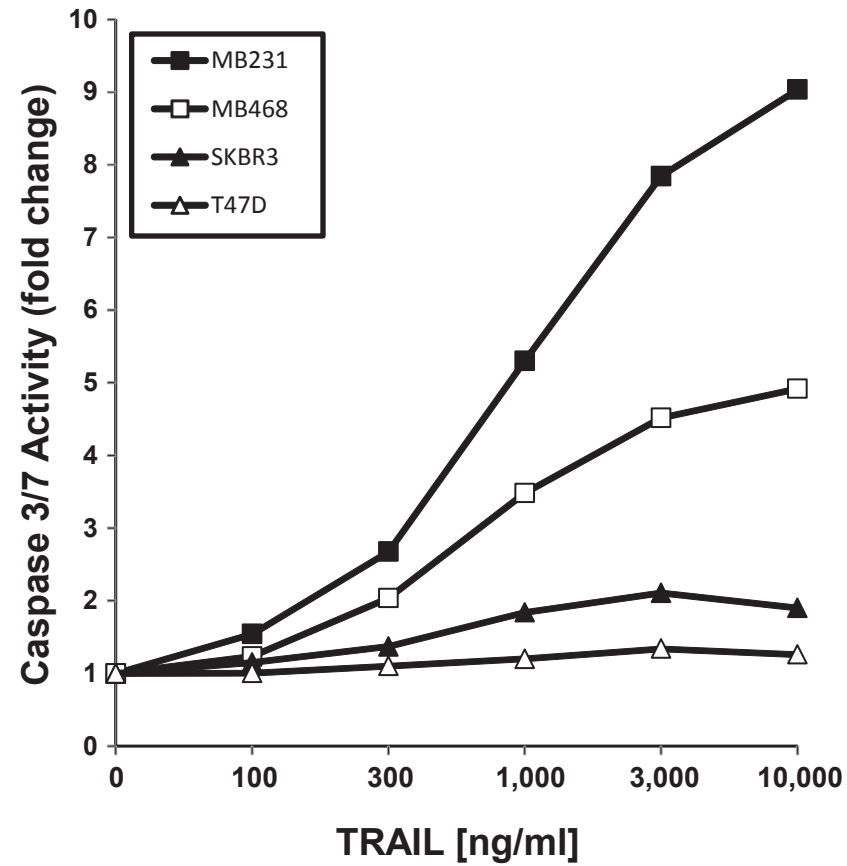

Supplement: Additional file 6: Figure S3 — Validation of genes identified by interaction analysis. Caspase-8 and caspase-3/7 activation, and cell viability in the absence (empty bars) and presence (black bars) of TRAIL for four siRNAs (A-D) corresponding to the genes shown. Mean data (±1 standard deviation) for control siRNA (siNeg) transfected cells are shown in each graph. The dashed line indicates the relevant 1 SD value for each assay, and the red dots indicate those siRNAs inducing fold-changes greater than 1 SD. [file bcr3645-S6.pdf]

**Figure S5**

**A**

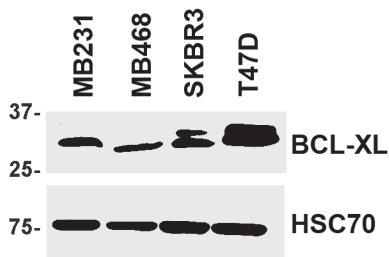

**B**

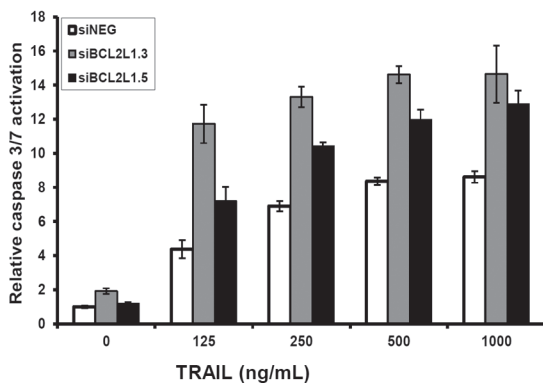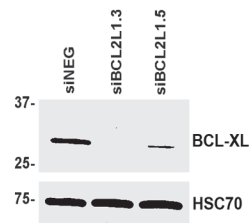

**C**

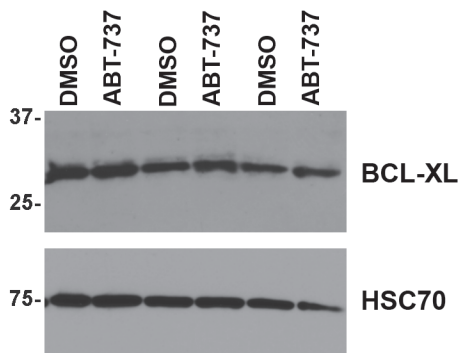

Supplement: Additional file 8: Table S4 — Caspase-3/7 siRNA secondary screen in a panel of breast cancer cell lines in the absence and presence of TRAIL. Data are for four different siRNAs per gene, shown as fold-change relative to siNeg-transfected cells in the absence of TRAIL. Values indicated in red are >2 SDs higher than TRAIL-induced caspase-3/7 in siNeg-treated cells. Values indicated in blue are >1 SD higher than TRAIL-induced caspase-3/7 in control siRNA (siNeg)-treated cells. [file bcr3645-S8.pdf]
