# Supplementary material for: Identification of novel molecular regulators of tumor necrosis factor-related apoptosis-inducing ligand (TRAIL)-induced apoptosis in breast cancer cells by RNAi screening
Source: Breast Cancer Res. 2014 Apr 17;16(2):R41. doi: 10.1186/bcr3645 (PMC4053258; doi:10.1186/bcr3645)
Supplement: Additional file 3: Figure S1 — Caspase-3/7 and cell-viability RNAi screens of the phosphatome and TRAIL-induced apoptosis in MB231 cells. (A) Controls included in the RNAi screens of the phosphatome gene set in MB231 cells for (i) caspase-3/7 activation and (ii) cell viability in the absence (white bars) or presence (black bars) of TRAIL. Cells were siRNA-transfected, treated with TRAIL, and assessed for caspase-3/7 activation and cell viability, as described in Figure 2. Data are normalized to the mean value of siNeg-transfected cells in the absence of TRAIL and are shown as the mean and standard deviation for each group. Comparison of TRAIL treated with siNeg-transfected untreated cells demonstrated a significant increase in caspase-3/7 activation and a significant decrease in viability. siCASP8 reduced caspase-3/7 activation (P = 1.0 × 10^-14^) and increased viability (P = 1.5 × 10^-14^) compared with siNeg-transfected cells. siFLIP increased caspase-3/7 (P = 1 × 10^-8^) activation and decreased viability (P = 7.0 × 10^-7^) compared with siNeg-transfected cells. ***P < 0.001. (B) To assess further the sensitivity of our assays, we confirmed that silencing Polo-like kinase 1 (PLK1), an essential protein in many cell lines, activated caspase-8, caspase-3/7, and decreased cell viability. The dashed line indicates the 1-SD value for each assay; the red dots indicate those siRNAs inducing fold-changes greater than 1 SD. (C) Identification of putative positive regulators of TRAIL-induced apoptosis. Genes for which three or four siRNAs decreased activation of caspase-3/7 in the presence of TRAIL 1 or more SDs over that observed in siNeg-transfected cells plus TRAIL were considered as putative positive regulators of TRAIL-induced apoptosis. The dashed line indicates the 1-SD fold-change, and a vertical line joins those siRNAs that induced at least this level of change for each gene. Data for the control siRNAs, siNeg, siCASP8, and siFLIP are included for reference. [file bcr3645-S3.pdf]

Garimella et al., Table S3

		Z' siNeg vs siCelldeath	Z' siNeg vs siCelldeath	Z' siFLIP No TRAIL vs. TRAIL	Z' siFLIP
		No TRAIL	PLUS TRAIL	No TRAIL vs. TRAIL	No TRAIL vs. TRAIL
Screen	Plate	Cell viability	Cell viability	Caspase 3/7	Caspase 8
Kinome gene set plus additional gene set	Plate 1	0.64	0.54	0.35	N/A
	Plate 2	0.76	0.69	0.63	0.09
	Plate 3	0.79	0.67	0.71	0.20
	Plate 4	0.74	0.63	0.24	0.26
	Plate 5	0.71	0.73	0.61	0.32
	Plate 6	0.36	0.67	0.78	0.54
	Plate 7	0.58	0.68	0.29	0.33
	Plate 8	0.54	0.72	0.74	0.21
	Plate 9	0.41	0.55	0.66	0.25
	Plate 10	0.53	0.61	0.57	0.32
	Plate 11	0.73	0.55	0.35	0.23
	Plate 12	0.69	0.66	0.15	0.40
Phosphatome gene set	Plate 1	0.35	0.20	0.65	-
	Plate 2	0.22	0.18	0.70	-
	Plate 3	N/A	0.77	0.78	-
	Plate 4	N/A	0.31	0.73	-

**Z'**	**Interpretation**
Between 0.5 and 1.0	An excellent assay
Between 0 and 0.5	A less robust assay
