# Supplementary material for: Identification of novel molecular regulators of tumor necrosis factor-related apoptosis-inducing ligand (TRAIL)-induced apoptosis in breast cancer cells by RNAi screening
Source: Breast Cancer Res. 2014 Apr 17;16(2):R41. doi: 10.1186/bcr3645 (PMC4053258; doi:10.1186/bcr3645)
Supplement: Additional file 7: Figure S4 — Caspase-3/7 activation by TRAIL in breast cancer cell lines. Cell lines were treated with increasing concentrations of TRAIL, as indicated along the X axis for 1 hour, and caspase-3/7 activation was measured by Caspase-Glo-3/7 assay, as described earlier. [file bcr3645-S7.pdf]

Garimella et al., Table S4

Caspase 3/7 Assay (Fold change relative to siNegative transfected cells, No TRAIL)

MB231 (TNBC-BASAL B)									T47D (ER Positive)							
	NO TRAIL				TRAIL					NO TRAIL				TRAIL		
	Mean		StDev		Mean		StDev									
siNeg	1.00		0.31		12.11		2.40		siNeg	1.00		0.10		1.78		0.30
siCASP8	1.04		0.21		2.30		0.63		siCASP8	0.94		0.08		1.02		0.26
siFLIP	1.27		0.27		18.71		2.16		siFLIP	0.98		0.11		1.26		0.20
GENE/siRNA	1	2	3	4	1	2	3	4	GENE/siRNA	1	2	3	4	1	2	3	4
BCL2L1	3.45	1.72	2.94	1.39	**80.88**	**39.30**	**31.46**	**22.06**	BCL2L1	3.05	1.32	1.22	0.98	**6.00**	**3.91**	**4.92**	**6.15**
BIRC2	1.71	3.15	1.90	2.36	**58.46**	**38.27**	**23.13**	**17.11**	BIRC2	0.87	1.02	1.03	1.02	**3.83**	**2.33**	**2.73**	**2.18**
CNKSR1	0.91	0.75	1.50	0.69	**51.54**	**36.50**	**24.17**	**12.50**	CNKSR1	0.94	0.89	0.85	0.85	**3.28**	**6.36**	1.71	1.80
MKNK1	1.02	1.31	0.91	0.81	**47.02**	**23.01**	**15.17**	**7.44**	MKNK1	0.93	1.02	0.95	0.94	1.38	**5.28**	**3.50**	1.86
BCR	0.89	1.88	0.79	1.14	**44.33**	**36.15**	**35.54**	**25.83**	BCR	1.11	1.08	1.11	0.93	**3.95**	**3.60**	**2.14**	**5.72**
PDPK1	1.12	1.05	1.05	1.88	**40.51**	**26.36**	**17.66**	**12.72**	PDPK1	1.00	0.78	0.95	0.93	**4.02**	**2.29**	**2.06**	**8.74**
PIP5K1C	1.01	1.40	1.25	0.88	**39.04**	**28.34**	**35.19**	**20.28**	PIP5K1C	1.02	1.16	0.99	1.46	**2.37**	**2.24**	**2.84**	1.69
FGFR4	1.17	0.84	0.82	0.80	**37.24**	**25.10**	**24.27**	**12.33**	FGFR4	0.99	1.00	1.05	0.90	**2.30**	**5.57**	**5.01**	1.56
ATP5A1	1.37	0.84	1.07	0.85	**36.56**	**31.29**	**27.78**	**17.28**	ATP5A1	1.48	1.01	0.90	1.12	**5.87**	**8.59**	**2.93**	**2.43**
SRC	1.45	0.84	1.23	1.01	**35.23**	**25.62**	**11.94**	**18.79**	SRC	1.01	1.11	1.15	0.75	**4.03**	**4.79**	**2.34**	1.36
ACTN4	1.00	1.44	0.94	1.27	**33.06**	**26.34**	**24.13**	**23.36**	ACTN4	0.89	0.98	0.90	0.81	**2.93**	**2.22**	**4.28**	**2.83**
PLK3	0.95	1.07	1.58	1.11	**31.57**	**30.63**	**29.29**	**13.62**	PLK3	1.01	1.06	0.93	1.15	**3.23**	**2.22**	1.26	1.81
PRKCI	1.00	0.78	0.94	1.29	**29.01**	**25.22**	**17.45**	**16.99**	PRKCI	1.15	1.19	0.95	1.02	**2.67**	2.00	1.68	**3.72**
RIOK3	1.34	1.28	1.17	0.94	**26.19**	**24.92**	**23.51**	**16.72**	RIOK3	0.99	1.03	1.32	1.02	**2.08**	**2.12**	2.03	**2.15**
HIPK2	0.76	0.78	0.88	0.78	**23.11**	**19.69**	**15.20**	8.68	HIPK2	1.10	0.91	0.89	0.90	1.56	**2.75**	**2.22**	1.08
IKBKB	1.09	0.93	1.35	0.81	**19.73**	**14.55**	13.70	9.69	IKBKB	1.04	1.23	0.91	0.87	1.98	1.91	1.10	1.62

MB468 (TNBC-BASAL A)									SKBR3 (HER2 amplified)							
	NO TRAIL				TRAIL					NO TRAIL				TRAIL		
siNeg	1.00		0.08		4.56		0.08		siNeg	1.00		0.12		1.21		0.19
siCASP8	0.87		0.08		1.51		0.41		siCASP	1.03		0.19		0.85		0.16
siFLIP	1.19		0.10		8.53		1.07		siFLIP	1.07		0.11		1.37		0.26
GENE/siRNA	1	2	3	4	1	2	3	4	GENE/siRNA	1	2	3	4	1	2	3	4
BCL2L1	4.84	0.98	1.65	1.08	**11.24**	**6.16**	**6.74**	4.56	BCL2L1	1.13	1.11	0.97	1.17	**3.70**	**1.44**	**1.48**	**1.46**
BIRC2	2.46	1.85	1.31	1.52	**9.32**	**11.05**	**5.67**	**6.31**	BIRC2	1.07	1.01	1.15	1.53	**1.96**	1.09	**1.78**	**2.00**
CNKSR1	1.53	0.71	0.77	0.98	**9.07**	**8.64**	**6.57**	**6.09**	CNKSR1	1.12	1.41	1.05	0.80	0.85	1.06	0.81	0.99
MKNK1	2.55	2.44	1.82	0.76	**8.86**	**8.15**	**9.08**	1.85	MKNK1	0.85	1.04	1.39	1.05	**1.60**	1.04	1.16	0.80
BCR	1.48	1.61	1.01	1.13	**10.70**	**8.10**	**5.15**	**9.83**	BCR	0.99	1.32	1.05	1.16	0.86	1.17	**1.46**	**1.80**
PDPK1	1.22	1.61	1.10	1.85	**8.38**	**8.25**	**6.69**	**7.63**	PDPK1	1.31	1.60	0.73	1.01	**1.90**	1.34	1.30	**1.98**
PIP5K1C	1.10	2.41	2.65	1.35	**6.66**	**7.60**	**10.26**	**6.19**	PIP5K1C	0.91	0.98	1.35	1.33	0.88	1.30	**1.49**	1.10
FGFR4	4.22	1.11	2.91	0.99	**8.35**	**8.19**	**6.94**	**5.85**	FGFR4	1.20	1.21	1.05	1.18	**1.57**	**1.95**	1.26	1.13
ATP5A1	1.81	1.88	1.45	1.22	**6.14**	**9.89**	**8.38**	**9.82**	ATP5A1	1.29	0.85	1.34	1.05	1.39	**2.06**	1.18	1.06
SRC	1.21	0.89	0.98	0.97	**10.10**	**8.90**	4.54	**8.36**	SRC	1.10	0.83	0.78	1.03	**1.43**	0.92	0.89	0.79
ACTN4	1.89	1.98	1.06	1.47	**8.84**	**19.58**	**11.78**	**8.26**	ACTN4	1.10	1.07	0.89	0.85	**1.75**	**2.06**	1.25	**1.59**
PLK3	2.19	1.50	2.13	1.60	**11.06**	**10.09**	**5.85**	4.26	PLK3	1.14	0.90	1.08	0.87	**1.92**	1.05	0.94	1.04
PRKCI	1.20	1.79	1.05	1.84	**8.51**	4.45	**7.42**	**7.17**	PRKCI	0.69	0.88	1.20	1.12	1.29	1.15	1.26	**1.97**
RIOK3	1.10	1.48	1.12	1.36	**6.77**	**10.02**	**5.43**	**9.07**	RIOK3	1.33	1.06	0.89	1.04	1.02	1.10	**1.46**	1.30
HIPK2	1.20	1.17	1.14	1.20	**8.86**	**5.80**	**6.96**	**9.60**	HIPK2	1.24	0.76	0.99	1.25	1.25	0.92	1.36	1.50
IKBKB	0.93	0.81	1.92	1.08	**5.84**	**3.96**	**6.47**	**4.90**	IKBKB	1.05	1.00	1.13	0.91	1.19	1.09	1.26	1.05
